# Endothelial adenosine receptor 2A loss alleviates diabetic vascular calcification by blocking CREB1-SNAI1-driven EndMT^☆^

**DOI:** 10.1016/j.phrs.2025.107981

**Published:** 2025-10-09

**Authors:** Yaqi Zhou, Dingwei Zhao, Qian Ma, Jiean Xu, Yongfeng Cai, Qiuhua Yang, Qingen Da, Kian Sheridan, Chunxiang Zhang, Clint L. Miller, Rajeev Malhotra, Suowen Xu, Mei Hong, Yuqing Huo

**Affiliations:** a State Key Laboratory of Chemical Oncogenomics, Key Laboratory of Chemical Genomics, School of Chemical Biology and Biotechnology, Peking University Shenzhen Graduate School, Shenzhen 518055, China; b Department of Physiology, Research Center of Basic Integrative Medicine, School of Basic Medical Sciences, Guangzhou University of Chinese Medicine, 232 Waihuan East Road, University Town, Guangzhou 510006, China; c Vascular Biology Center, Department of Cellular Biology and Anatomy, Medical College of Georgia, Augusta University, Augusta, GA 30912, USA; d Departments of Ophthalmology, Medicine and Molecular and Cellular Biology, Baylor College of Medicine, Houston, TX 77030, USA; e Department of Pharmacological Sciences, Stony Brook University, Stony Brook, NY 11794, USA; f Emory University, Atlanta, GA 30322, USA; g Department of Cardiology, Key Laboratory of Medical Electrophysiology, Ministry of Education, Institute of Cardiovascular Research, The Affiliated Hospital of Southwest Medical University, Southwest Medical University, Luzhou 646000, China; h Department of Biochemistry and Molecular Genetics, University of Virginia, Charlottesville, VA 22908, USA; i Center for Public Health Genomics, University of Virginia, Charlottesville, VA 22908, USA; j Cardiovascular Research Center, Division of Cardiology, Department of Medicine, Massachusetts General Hospital, Harvard Medical School, Boston, MA 02114, USA; k Department of Endocrinology, Centre for Leading Medicine and Advanced Technologies of IHM, The First Affiliated Hospital of USTC, Division of Life Sciences and Medicine, University of Science and Technology of China, Hefei 230001, China; l Anhui Provincial Key Laboratory of Metabolic Health and Panvascular Diseases, Hefei, 230001, China; m Institute of Endocrine and Metabolic Diseases, University of Science and Technology of China, Hefei 230001, China

**Keywords:** Adenosine receptor 2 A, Vascular calcification, Endothelial-to-mesenchymal transition, Diabetes mellitus, CREB1, SNAI1

## Abstract

Vascular calcification (VC), a common complication associated with diabetes mellitus (DM), substantially increases the risk of cardiovascular diseases and is associated with elevated mortality in individuals with DM. Endothelial-to-mesenchymal transition (EndMT) imparts phenotypic plasticity to vascular endothelial cells (VECs), granting them the potential for osteogenic differentiation, which is a crucial mechanism in regulating VC. Notably, adenosine-ADORA2A-mediated endothelial dysfunction plays a pivotal regulatory role in cardiovascular diseases. However, the specific role of endothelial ADORA2A in diabetic VC remains to be elucidated. In this study, we found that ADORA2A was upregulated in the endothelium of diabetic mice and cultured human aortic endothelial cells (HAECs) with high glucose treatment. Deletion of endothelial *Adora2a* or pharmacologic inhibition of ADORA2A with KW6002 attenuated EndMT, osteogenic differentiation, and calcium deposit in diabetic aortas of *Ins2*^Akita/+^ mice. Consistently, ADORA2A knockdown or inhibition in HAECs suppressed EndMT and osteogenic differentiation in response to high glucose and other pro-calcified conditions. Mechanistically, *ADORA2A* induced HAECs to undergo EndMT and osteogenic differentiation by regulating the CREB1-SNAI1 axis. Collectively, our results reveal a previously unrecognized role of endothelial ADORA2A inactivation in attenuating diabetic VC via modulation of EndMT. These insights offer a compelling mechanistic rationale for leveraging ADORA2A antagonists as promising therapeutic agents against diabetic VC.

## 1. Introduction

Cardiovascular disease (CVD) is a frequent complication and the leading cause of morbidity and mortality in patients with diabetes mellitus (DM) [1]. Vascular calcification (VC), characterized as the accumulation of hydroxyapatite and bone-like tissue in the arterial walls, is a critical predictor of diabetes-associated cardiovascular events such as retinopathy, nephropathy, stroke, and critical limb ischemia[2,3]. The key contributing factors that predispose individuals with DM to VC include hyperglycemia, oxidative stress, lipid metabolism disorders, inflammation, and dysregulation of bone regulatory proteins [4]. Current therapies for VC mainly aim to control these underlying factors; however, their effectiveness remains limited, and significant challenges persist. Once VC occurs, it is difficult to reverse the process [5]. Despite the considerable impact of VC in DM, the underlying mechanism remains elusive. Further research is necessary to develop new effective strategies for preventing or treating VC in diabetic patients.

VC occurs through synergistic crosstalk between various cell types and cytokines [6]. Despite the complexity of this process, osteoblast-like cells ultimately synthesize and mineralize the calcified extracellular matrix [7]. Several cell types, including vascular endothelial cells (VECs), vascular smooth muscle cells (VSMCs), pericytes, macrophages, and progenitor cells, have been implicated in regulating the progression of VC[8,9]. Although all these cell types may contribute to the pool of calcifying cells, their relative contributions remain unclear and may vary depending on the pathological context. Nonetheless, the initiation of these chronic pathological changes is often triggered by endothelial dysfunction, highlighting the critical role of VECs in this process [10,11]. VECs facilitate the differentiation into osteoblast-like cells and acquire multipotency through endothelial-mesenchymal transitions (EndMT), which have been demonstrated to promote arterial calcification in DM [12–14]. EndMT is a progressive process wherein ECs gradually lose their morphological and functional characteristics, while gaining mesenchymal traits such as proliferation, migration, and collagen synthesis. High-glucose stimulation significantly upregulates the expression of mesenchymal cell-specific markers and promotes the occurrence of EndMT in cultured human aortic endothelial cells (HAECs) [15]. Furthermore, previous research has demonstrated that VECs serve as a source of osteoblast-like cells in calcified vessels of diabetic mice [12], underscoring the crucial role of EndMT-mediated osteogenic differentiation of VECs in the progression of diabetic VC. Therefore, the blockade of EndMT may provide a promising therapeutic avenue for the treatment of diabetic VC.

Adenosine is a ubiquitous endogenous nucleoside that exerts broad physiological functions through its interaction with adenosine receptors (ARs), members of the G protein-coupled receptors (GPCRs) superfamily [16]. Given that approximately 40 % of modern pharmaceuticals target GPCRs, modulating the activity of ARs holds considerable promise for clinical intervention. There are four subtypes of ARs—adenosine receptor 1 (ADORA1), adenosine receptor 2 A (ADORA2A), adenosine receptor 2B (ADORA2B), and adenosine receptor 3 (ADORA3)—each with distinct tissue distributions and regulatory functions in mammals. Previous studies have provided compelling evidence that adenosine and its receptor ADORA2A are involved in the regulation of collagen synthesis in fibroblasts and the development of tissue fibrosis [17–21], while EndMT is recognized as a major source of myofibroblasts during fibrotic processes [22,23]. These findings imply that the adenosine-ADORA2A system may play a regulatory role in EndMT. However, the specific involvement of ADORA2A in EndMT has not yet been identified.

In this study, we used both *in vitro* and *in vivo* models of diabetic VC to investigate whether ADORA2A is required for the induction of EndMT and examined the role of ADORA2A inhibitor in the development of DM-associated VC.

## 2. Materials and methods

### 2.1. Animals

Heterozygous *Ins2*^Akita^ (*Ins2*^Akita/+^) mice were purchased from the Jackson Laboratory (Cat# 003548, the Jackson Laboratory). *Adora2a*-^Flox/Flox^ (*Adora2a*^F/F^) mice were kindly gifted by Dr. Joel Linden (La Jolla Institute for Allergy and Immunology, CA, USA). Mice with *Adora2a* deficiency in endothelial cells (*Adora2a*^ΔVEC^) were obtained by cross-breeding the *Cdh5*-Cre (*Cdh5*^Cre^) transgenic line (Cat# 006137, the Jackson Laboratory, ME, USA) with *Adora2a*^F/F^ mice. All mice used in this study were on a C57BL/6 J background. Animals were housed in groups and maintained under the following conditions: temperature of 23 ± 2°C, relative humidity of 60 ± 10 %, and a 12/12 h light/dark cycle with free access to food and water. Genotyping of these mice was performed using PCR amplification with tail DNA samples. The sequences of detailed specific primers used for genotyping analysis are listed as follows: *Ins2*^Akita/+^ (5’-TGCTGATGCCCTGGCCTGCT-3’ and 5’-TGGTCCCACATATGCACATG-3’), *Adora2a*^F/F^ (5’-GGGCAAGATGGGAG TCATT-3’ and 5’-ATTCTGCATCTCCCGAAACC-3’), and *Cdh5*^Cre^ (5’-GCGGTCTGGCAGTAAAAACTATC-3’ and 5’-GTGAAACAGCATTGC TGTCACTT-3’).

### 2.2. Mouse model of diabetic vascular calcification

To investigate the progression of diabetic vascular calcification, we employed male *Ins2*^Akita/+^ mice, a well-established monogenic model of type I DM. These mice carry a spontaneous point mutation in the *Insulin 2* (*Ins2*) gene, which causes insulin misfolding, endoplasmic reticulum stress, β-cell dysfunction, and reduced insulin secretion. These mice develop insulin-dependent diabetes, characterized by persistent hyperglycemia and marked weight loss beginning at 4–8 weeks of age, which is consistent with our observations from monitoring the body weight and blood glucose levels in *Ins2*^Akita/+^ mice from 4 to 40 weeks of age using the method described previously [24] (Supplementary Figure 1 A, Supplementary Figure 3 A and 3B). The phenotype is more severe in males than females and occurs without obesity or insulitis, distinguishing this model from autoimmune or obesity-related diabetes models.

Given their stable hyperglycemia and absence of confounding metabolic or inflammatory factors, *Ins2*^Akita/+^ mice are well-suited for studying hyperglycemia-driven vascular pathology, including diabetic macrovascular complications such as VC. This model is particularly valuable for investigating molecular pathways and therapeutic targets like ADORA2A involved in diabetic vasculopathy.

### 2.3. Mouse model of vascular calcification within CKD

A murine model of chronic kidney disease (CKD) was generated via a two-stage surgical procedure involving 5/6th nephrectomy and ligation of renal poles. In the initial procedure, mice were anesthetized with isoflurane, and the upper and lower poles of the left kidney were constricted using 3–0 silk sutures with appropriate tension. One week later, a second surgery was conducted to excise the right kidney by ligating and transecting the renal vasculature. In contrast, control animals subjected to sham surgery underwent comparable operative exposure without vessel ligation or kidney removal.

To induce vascular calcification, mice were placed on a high-phosphate diet (1.8 %; Guangdong Medical Laboratory Animal Center, Guangdong, China) starting one week after recovery. In addition, cholecalciferol (Cat# C9756, Sigma-Aldrich, MO, USA) was administered via oral gavage at a dose of 1 μg/kg, three times weekly, for four weeks. In contrast, animals in the sham-operated group received a standard phosphate diet containing 0.8 % phosphate.

### 2.4. Istradefylline (KW6002) administration

To assess the *in vivo* efficacy of the ADORA2A antagonist istradefylline (KW6002), *Ins2*^Akita/+^ mice were randomly assigned to receive either KW6002 or vehicle. Daily intraperitoneal injections of KW6002 (10 mg/kg) or vehicle were administered from 32 to 40 weeks of age. Littermate wild-type (WT) mice treated with vehicle served as negative controls. The selected dosage of KW6002 has been shown to inhibit ADORA2A-mediated responses in established rodent disease models selectively [25].

### 2.5. En face staining of aortic endothelium

Mice were euthanized and subsequently perfused with PBS, followed by 4 % paraformaldehyde for tissue fixation. The aortas were carefully dissected and further fixed in 4 % paraformaldehyde at room temperature. Tissues were permeabilized in PBS containing 0.5 % Triton X-100 for 30 min, then incubated with 10 % normal goat serum for 1 h to block nonspecific binding. Aortas were incubated overnight at 4°C with primary antibodies against mouse ADORA2A (5 μg/mL; Cat# 05–717, Millipore, MA, USA) and CD31 (4 μg/mL; Cat# DIA-310, Dianova, Hamburg, Germany). The following day, samples were incubated for 1 h at room temperature with secondary antibodies: Alexa Fluor 594 goat anti-mouse IgG (10 μg/mL, Cat# ab150116, Abcam, Cambridge, UK) and Alexa Fluor 488 goat anti-rat IgG (10 μg/mL, Cat# ab150157, Abcam, Cambridge, UK). Nuclei were counterstained using DAPI (Cat# D1306, Invitrogen, CA, USA). Finally, aortas were flat-mounted onto microscope slides using fluorescence mounting medium (Cat# H-1000, Vector Laboratories, CA, USA), and immunofluorescent signals were visualized using a confocal microscope (Nikon, Tokyo, Japan).

### 2.6. Determination of arterial calcification

Arterial calcification was assessed using alizarin red and von Kossa staining methods. Mouse aortic segments were fixed in 4 % paraformaldehyde and embedded in O.C.T. compound. Tissue sections of 6 μm thickness were prepared for staining. For alizarin red staining, slides were incubated with 0.2 % alizarin red solution (pH 8.3; Cat# C0140–100 mL, Beyotime, Shanghai, China) at room temperature for 20 min, followed by thorough rinsing with deionized water to remove excess dye. Von Kossa staining involved exposing sections to 1 % silver nitrate solution (Cat# GMS80045.3, Genmed, Shanghai, China) under a 60-watt light source for 45 min. Remaining silver deposits were removed by treatment with 5 % sodium thiosulfate for 5 min, after which the sections were counterstained with nuclear fast red (Cat# GMS40011, Genmed, Shanghai, China) according to the manufacturer’s instructions. Stained sections were imaged using an inverted microscope.

For quantitative analysis of calcium deposition, descending aortas were excised, freeze-dried to constant weight, and decalcified in 0.6 mM hydrochloric acid at 37°C for 48 h. The calcium concentration in the supernatant was determined colorimetrically using the QuantiChrom^™^ Calcium Assay Kit (Cat# DICA-500, BioAssay Systems, CA, USA). Calcium content was normalized to the dry tissue weight and expressed as fold change relative to the control group.

### 2.7. Hematoxylin and eosin (HE) staining

Hematoxylin and eosin (HE) staining was performed on frozen sections of descending aortas. Tissue samples embedded in O.C.T. compound were sectioned at 6 μm thickness. Slides were first rinsed with PBS before being immersed in hematoxylin solution (Cat# 22050111, Thermo Fisher Scientific, MA, US) for 3 min. After a brief water wash, sections were sequentially dehydrated using 70 % and 90 % ethanol solutions. Subsequently, eosin (Cat# 22050110, Thermo Fisher Scientific, MA, US) staining was applied for 1 min, followed by further dehydration steps with graded alcohol and xylene. Finally, coverslips were mounted using neutral resin. Stained sections were visualized under an inverted microscope for imaging.

### 2.8. Immunofluorescent staining

Frozen sections of O.C.T. compound-embedded samples were prepared at a thickness of 6 μm. After washing with PBS, the slides were fixed in 4 % paraformaldehyde for 15 min, followed by blocking with 10 % normal goat serum for 1 h. The sections were then incubated overnight at 4°C with primary antibodies targeting RUNX2 (5 μg/mL; Cat# 192256, Abcam, Cambridge, UK) and CD31 (4 μg/mL; Cat# DIA-310, Dianova, Hamburg, Germany). Subsequently, a mixture of Alexa Fluor 594 goat anti-rabbit IgG (10 μg/mL, Cat# ab150080, Abcam, Cambridge, UK) and Alexa Fluor 488 goat anti-rat IgG (10 μg/mL, Cat# ab150157, Abcam, Cambridge, UK) was applied for 1 h at room temperature. Nuclear staining was performed using DAPI (Cat# D1306, Invitrogen, CA, USA). Finally, slides were mounted with anti-fade mounting medium (Cat# H-1000, Vector Laboratories, CA, USA), and imaging was conducted using confocal microscopy (Nikon, Tokyo, Japan).

### 2.9. Human aortic endothelial cells (HAECs) culture and treatment

Primary human aortic endothelial cells (HAECs) were obtained from American Type Culture Collection (Cat# PCS-100–011, ATCC, VA, USA) and cultured in endothelial growth medium (EGM-2, Cat# CC-3162, Lonza, MD, USA) in a humidified atmosphere with 5 % CO_2_ in a 37°C humidified incubator. The culture medium was refreshed every two days until cell confluence reached approximately 80 %. For all *in vitro* assays, HAECs at passages 3–5 were utilized. HAECs were subjected to treatment with either high glucose (HG; 25 mM D-glucose), normal glucose (NG; 5.5 mM D-glucose supplemented with 19.5 mM mannitol as an osmotic control), or standard growth medium (Blank; 5.5 mM glucose) for the specified durations. In some experimental groups, HAECs were co-incubated with ADORA2A antagonist KW6002 (100 nM).

### 2.10. Mouse aortic endothelial cells (MAECs) isolation and culture

Primary mouse aortic endothelial cells (MAECs) were derived from 3-week-old mice. Briefly, type I mouse tail collagen (Cat# 354236, BD, NJ, USA) was prepared by mixing with high-glucose Dulbecco’s Modified Eagle Medium (DMEM; Cat# SH30021.01, Hyclone, UT, USA) supplemented with 10 % fetal bovine serum (FBS), followed by pH adjustment using 0.1 M sodium hydroxide. The collagen solution was dispensed into 24-well plates (500 μL/well) and allowed to polymerize at 37°C for 1 h. Subsequently, wells were preconditioned overnight in MAEC-specific medium, consisting of EGM-2 supplemented with 10 % FBS, 1 % Antibiotic-Antimycotic, and 0.15 g of cyclic adenosine monophosphate (cAMP) (Cat# A9501, Sigma-Aldrich, MO, USA). After the mice were euthanized by CO_2_ asphyxiation and cervical dislocation, the aortas were carefully dissected after *in situ* perfusion with sterile PBS. Descending aortas were collected and washed with PBS. Aortic segments were placed endothelium-side down onto type I mouse tail collagen-coated wells and incubated at 37°C for 48 h to promote endothelial cell migration. After tissue removal, fresh MAECs culture medium was added, and the cells were incubated for an additional 24 h. Cells were then washed with PBS and enzymatically detached using 0.3 % collagenase D (Cat# 11088866001, Roche, Mannheim, Germany) at 37°C for 20 min. The cell suspension was collected into a tube and centrifuged at 1000 rpm for 3 min. The cell pellet was resuspended in MAECs culture medium and transferred to T25 flasks coated with 2 % gelatin (Cat# G1393, Sigma-Aldrich, MO, USA). Cells were plated at a density depending on cell recovery and cultured in a humidified incubator with 5 % CO_2_ at 37°C.

### 2.11. Small interfering RNA (siRNA) interference of HAECs

HAECs were cultured in EGM-2 medium until they reached approximately 60–70 % confluence in 6-well culture plates. For gene silencing, cells were transfected with 50 nM of small interfering RNA (siRNA) targeting human *ADORA2A* (Cat# sc-39850, Santa Cruz Biotechnology, TX, USA), human *MGP* (Cat# sc-44626, Santa Cruz Biotechnology, TX, USA), human cAMP response element-binding protein 1 (*CREB1)* (Cat# sc-29281, Santa Cruz Biotechnology, TX, USA), or human snail family transcriptional repressor 1 (*SNAI1)* (Cat# sc-38398, Santa Cruz Biotechnology, TX, USA) using Lipofectamine RNAiMAX Reagent (Cat# 13778–150, Invitrogen, CA, USA), following the manufacturer’s instructions. A non-targeting scrambled siRNA (si*CTRL*; Cat# sc-37007, Santa Cruz Biotechnology, TX, USA) was included as the negative control.

### 2.12. Osteogenic induction

HAECs were cultured in 12-well plates using EGM-2 until they reached approximately 60–70 % confluence. Cells were then transfected with either human *MGP* siRNA or a negative control siRNA at a concentration of 50 nM. On the following day, cells treated with si*MGP* were switched to calcified medium (CM), composed of EGM-2 supplemented with 10 % FBS, 1 % Antibiotic-Antimycotic, 10^−8^ M dexamethasone (Cat# D4902, Sigma-Aldrich, MO, USA), 0.2 mM L-ascorbic acid (Cat# 49752, Sigma-Aldrich, MO, USA), and 10 mM β-glycerophosphate (Cat# G9422, Sigma-Aldrich, MO, USA). In contrast, si*CTRL*-transfected cells were maintained in standard growth medium (GM), which consisted of EGM-2 containing 10 % FBS and 1 % Antibiotic-Antimycotic, serving as the negative control group. Both experimental and control groups were cultured for a duration of up to 14 days, with medium replacement occurring every two days throughout the induction period.

Mineral deposition was assessed by alizarin red staining. Cells were first fixed using 4 % paraformaldehyde and subsequently rinsed with deionized water. Next, they were incubated in 2 % alizarin red solution (pH 4.2; Cat# ECM-815, Sigma-Aldrich, MO, USA) at room temperature for 5 min, followed by thorough washing with deionized water to remove any unbound stain. Images of mineral deposition was then visualized and captured using an inverted microscope.

### 2.13. Adenovirus transduction of HAECs

HAECs grown to approximately 60 %−70 % confluence were transduced with either Ad-*Ctrl* or Ad-*ADORA2A* adenovirus by incubating the cells in 1 mL of endothelial basal medium (EBM-2; Cat# CC-3156, Lonza, MD, USA) for 1 h. Following viral exposure, the medium was replaced with fresh endothelial growth medium (EGM-2), and the cells were returned to standard culture conditions. Samples were harvested at specified time intervals for subsequent analyses.

### 2.14. Western blotting

For protein extraction, cultured cells and aortic tissues were lysed using RIPA buffer supplemented with protease inhibitor cocktail (Cat# 05892970001, Roche, Mannheim, Germany) and phenylmethylsulfonyl fluoride (PMSF; Cat# ST506, Beyotime, Shanghai, China). Homogenates were centrifuged at 12,000 × *g* for 10 min at 4°C, and the supernatants were harvested. Protein levels were assessed using the Pierce BCA Protein Assay Kit (Cat# 23225, Thermo Fisher Scientific, MA, US). Equal amounts of protein (10 μg per sample) were separated by 8–12 % SDS-PAGE and subsequently transferred to PVDF membranes. The membranes were incubated with relevant primary antibodies overnight at 4°C. The primary antibodies utilized included: ADORA2A (1 μg/mL; Cat# 05–717, Millipore, MA, USA), CDH5 (0.2 μg/mL; Cat# sc-9989, Santa Cruz Biotechnology, CA, USA), SOX2 (1:1000; Cat# ab97959, Abcam, Cambridge, UK), ALPL (0.55 μg/mL; Cat# 11187–1-AP, ProteinTech, IL, USA), RUNX2 (1:1000; Cat# 12556, Cell Signaling Technology, MA, USA), ACTA2 (0.1 μg/mL; Cat# sc-56499, Santa Cruz Biotechnology, CA, USA), TAGLN (0.5 μg/mL; Cat# ab14106, Abcam, Cambridge, UK), S100A4 (1:1000; Cat# ab197896, Abcam, Cambridge, UK), FN1 (0.5 μg/mL; Cat# ab2413, Abcam, Cambridge, UK), CREB1 (0.5 μg/mL; Cat# 9197, Cell Signaling Technology, MA, USA), phosphorylated CREB at Ser133 (0.5 μg/mL; Cat# 9198, Cell Signaling Technology, MA, USA), SNAI1 (1 μg/mL; Cat# ab180714, Abcam, Cambridge, UK), SP7 (1:1000; Cat# ab209484, Abcam, Cambridge, UK), BGLAP (1 μg/mL; Cat# ab93876, Abcam, Cambridge, UK), COL1A1 (1 μg/mL; Cat# NB600408, Novus Biologicals, CO, USA), and GAPDH (0.5 μg/mL; Cat# 5174S, Cell Signaling Technology, MA, USA). On the following day, membranes were washed thrice with the mixed solution of Tris-Buffered Saline and Tween 20 (TBST), then incubated with either anti-mouse IgG-HRP (1:2000; Cat# 7076, Cell Signaling Technology, MA, USA) or anti-rabbit IgG-HRP (1:2000; Cat# 7074, Cell Signaling Technology, MA, USA) for 1 h at room temperature. Subsequently, the membranes were washed again with TBST three times. Bands were visualized using enhanced chemiluminescence substrate (Cat# 1705060, Bio-Rad, CA, USA) and imaged with the ChemiDoc MP system (Bio-Rad, CA, USA). Densitometric analysis was performed using ImageJ software, with normalization to GAPDH as an internal reference.

### 2.15. Quantitative real-time PCR (qPCR) analysis

Total RNA from cultured cells was extracted with the TRIzol reagent (Cat# 15596018, Invitrogen, CA, USA) according to the manufacturer’s instructions. 1 μg of total RNA was reverse-transcribed into cDNA with TransScript^®^ One-Step gDNA Removal and cDNA Synthesis SuperMix kit (Cat# AT311, TransGen Biotech, Beijing, China). PCR amplification was performed via a Bio-rad CFX96 instrument (Bio-rad, CA, USA) with universal SYBR green mix (Cat# AQ601, TransGen Biotech, Beijing, China). The sequences of specific primers are listed in Supplemental Table 1. Quantification of relative gene expression was calculated with the 2^−ΔΔCT^ method using *18S* ribosomal RNA as the internal control.

### 2.16. Chromatin immunoprecipitation (ChIP) coupled with quantitative PCR

ChIP assays were conducted following the manufacturer’s protocol using the Pierce Agarose ChIP Kit (Cat# 26156, Thermo Fisher Scientific, MA, USA) on cross-linked HAECs at a density of 1 × 10^^^6 cells. Chromatin was sheared by sonication to generate DNA fragments between 200 and 1000 base pairs in length. Immunoprecipitation was performed using Protein G agarose beads to capture antibody-antigen complexes with antibodies specific to CREB1 (Cat# 9197S, Cell Signaling Technology, MA, USA) or control IgG antibody (Cat# 3900S, Cell Signaling Technology, MA, USA). To evaluate CREB1 binding enrichment on the *SNAI1* promoter, the immunoprecipitated DNA was analyzed by quantitative PCR. Each qPCR reaction (20 μL total volume) included 2 μL of DNA template and 100 nM of primers. The primers targeting the human *SNAI1* promoter region were as follows: forward 5’-ATCCCACCCCATCCCTGGAA-3’ and reverse 5’-ACGAAGTAAACAGATAATTACAATGCA-3’. ChIP signals were quantified and normalized relative to the percentage of input DNA to ensure accurate and reliable comparison of enrichment levels.

### 2.17. Dual-luciferase reporter assay

To construct the human *SNAI1* GLuc-ON promoter reporter clone, a 1294 bp fragment of the *SNAI1* gene promoter (−1225 to +68 relative to the transcription start site) was PCR-amplified and subcloned into the pEZX-PG04.1 vector (Cat# HPRM46456-PG04, GeneCopoeia, MD, USA) at multiple cloning sites (5’: *Bgl*II, *Eco*RI; 3’: *Hin*dIII), using secreted alkaline phosphatase (SEAP) as an internal control reporter. Dual-luciferase reporter assays were also performed with the negative control vector (Cat# NEG-PG04, GeneCopoeia, MD, USA) and the positive control vector (Cat# GAPDH-PG04, GeneCopoeia, MD, USA) as controls.

Cells with different treatments were used for the dual-luciferase reporter assay. HAECs were first transfected with Ad-*ADORA2A* or Ad-*CTRL* for 48 h, followed by pretreatment with SQ22536 (10 μM) for 24 h. HAECs were subsequently transfected with 100 ng of indicated constructed reporter plasmids (HPRM46456-PG04, NEG-PG04, and GAPDH-PG04) using Lipofectamine RNAiMAX Reagent (Cat# 13778–150, Invitrogen, CA, USA) and cultured for 48 h. Relative luciferase activity was measured using the Secrete-Pair Dual Luminescence Assay Kit (Cat # LF031, GeneCopoeia, MD, USA) according to the manufacturer’s instructions and was quantified using a luminometer as previously described [26].

### 2.18. Data and statistical analysis

Based on previous experience and preliminary data, we calculated the optimal animal numbers and sample sizes needed. For experiments involving C57BL/6 J WT mice, animals were randomly assigned to groups. Researchers for the outcome evaluations were blind to the treatment assignment. All experiments were performed on individual animals or independently cultured cells, with each experiment independently replicated a minimum of three times, and no outliers were removed from the data. All experimental quantitative data are expressed as mean ± standard error of the mean (SEM). Statistical analysis and graphing were performed using GraphPad Prism software (Version 9.0; La Jolla, CA, USA). For data that passed both the normality and variance tests, an unpaired Student’s *t*-test was used. For data that passed the normality test but failed the variance test, Brown-Forsythe and Welch’s ANOVA tests were used, followed by Dunnett’s T3 multiple comparison test. For data that passed both the normality and variance tests, one-way ANOVA was used, followed by Bonferroni’s *post hoc* test. The specific statistical test used for each experiment will be indicated in the figure legends. A *p*-value < 0.05 was considered statistically significant. Statistical significance is defined as follows: *p < 0.05, **p < 0.01, ***p < 0.001, and ****p < 0.0001. “ns” indicates no significant difference.

## 3. Results

### 3.1. Expression of ADORA2A is increased in both the endothelium of Ins2^Akita/+^ mice and cultured HAECs exposed to high glucose

The *Ins2*^Akita/+^ mice, a type of model I DM, were used to investigate the progression of diabetic calcific vasculopathy. A progressive notable elevation of blood glucose concentration in *Ins2*^Akita/+^ mice was observed (Supplementary Figure 1 A). To verify the aortic calcification in diabetic mice, we analyzed the expression of calcification-related markers in the aortas of *Ins2*^Akita/+^ mice at 40 weeks of age. The expression of multipotent marker sex determining region Y-box transcription factor 2 (SOX2), and osteogenic markers runt-related transcription factor 2 (RUNX2) and Osterix (SP7), were all upregulated in the diabetic arteries (Supplementary Figure 1B). Double immunofluorescence staining showed that hyperglycemia induced RUNX2 expression in the aortic tissues with disrupted endothelium (Supplementary Figure 1 C). Consistently, Alizarin red staining and von Kossa staining suggested severe calcification in the aortas of *Ins2*^Akita/+^ mice (Supplementary Figure 1D).

**Fig. 1.**
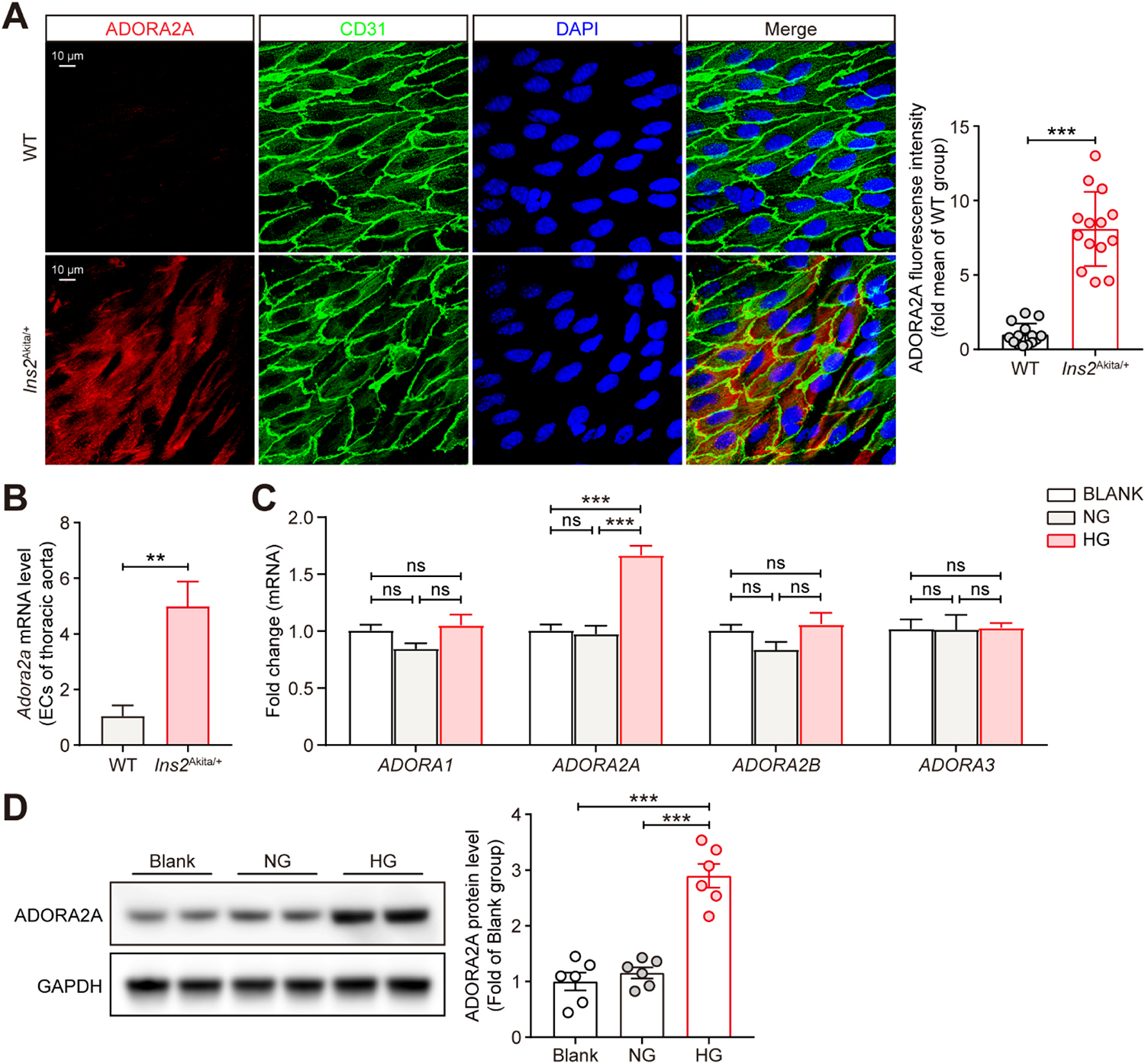
Increased expression of ADORA2A in both aortic ECs of *Ins2*^Akita/+^ mice and HAECs with HG treatment. **A**, Representative images of *en face* immunofluorescence staining and quantification data of ADORA2A (red) levels in thoracic aorta endothelium of *Ins2*^Akita/+^ mice (n = 14 mice per group). Endothelial cells were marked with CD31 (green). Nucleus (blue) was stained with DAPI. Scale bar is 10 μm. **B**, Real-time PCR analysis of mRNA levels of *Adora2a* in ECs of thoracic aortas in *Ins2*^Akita/+^ mice. n = 9 mice per group. One scatter data was obtained for every three mice samples. **C**, Real-time PCR analysis of mRNA expression for adenosine receptors (*ADORA1*, *ADORA2A*, *ADORA2B*, and *ADORA3*) in HAECs exposed to high glucose (HG; 25 mM) for 48 h. Mannitol (NG; 5.5 mM glucose and 19.5 mM mannitol) was used as a control for hyperosmolarity (n = 6). **D**, Western blot analysis and quantification data of protein levels of ADORA2A in HAECs exposed to high glucose for 72 h (n = 6). Data are represented as means ± SEM. Statistical significance was determined by unpaired Student’s *t*-test (A, B) and one-way ANOVA with the Bonferroni’s *post hoc* test (C, D). **p < 0.01, and ***p < 0.001 for indicated comparisons. “ns” indicates no significant difference.

To test whether endothelial ADORA2A was associated with diabetic VC, we first examined the levels of ADORA2A in arterial endothelium of *Ins2*^Akita/+^ mice at 20 weeks of age. *En face* staining of the endothelium showed enhanced expression of ADORA2A in the thoracic aorta endothelium of *Ins2*^Akita/+^ mice compared with WT mice (Fig. 1A), whereas calcification was not observable at this time point, indicating that increased ADORA2A may precede VC in DM. Furthermore, we found that ECs isolated from thoracic aortas of *Ins2*^Akita/+^ mice displayed remarkably increased mRNA levels of *Adora2a* (Fig. 1B), suggesting that increased endothelial ADORA2A expression may participate in the pathogenesis of VC in DM.

VECs have previously been shown to undergo EndMT to dedifferentiate into mesenchymal-like cells, which eventually contribute osteogenic cells to arterial calcification of diabetic mice. To determine the role of ADORA2A in regulating EndMT and osteogenesis, HAECs were incubated with high glucose to establish *in vitro* diabetic cell models and ADORA2A was increased at both mRNA and protein levels (Figs. 1C and 1D). This was consistent with those found in *Ins2*^Akita/+^ mice.

### 3.2. Endothelial Adora2a deficiency ameliorates aortic calcification in Ins2^Akita/+^ mice

Based on the above results, we hypothesized that inactivation of endothelial ADORA2A might have beneficial effects on the development of VC in *Ins2*^Akita/+^ mice. We first generated VEC-specific *Adora2a*-deficient mice (*Adora2a*^ΔVEC^) and their controls (*Adora2a*^WT^). The efficiency of *ADORA2A* knockout in VECs of aortas was proved by western blot analysis (Supplementary Figure 2A and 2B). Then, *Ins2*^Akita/+^ mice were bred with *Adora2a*^△VEC^ mice and the control *Adora2a*^WT^ mice to generate *Adora2a*^△VEC^ *Ins2*^Akita/+^ mice and *Adora2a*^WT^*Ins2*^Akita/+^ mice, respectively. Both *Adora2a*^△VEC^ *Ins2*^Akita/+^ mice and *Adora2a*^WT^ *Ins2*^Akita/+^ mice were exhibited aggravated blood glucose levels and severe body weight loss, but there was no difference between these two groups (Supplementary Figure 3A and 3B).

**Fig. 2.**
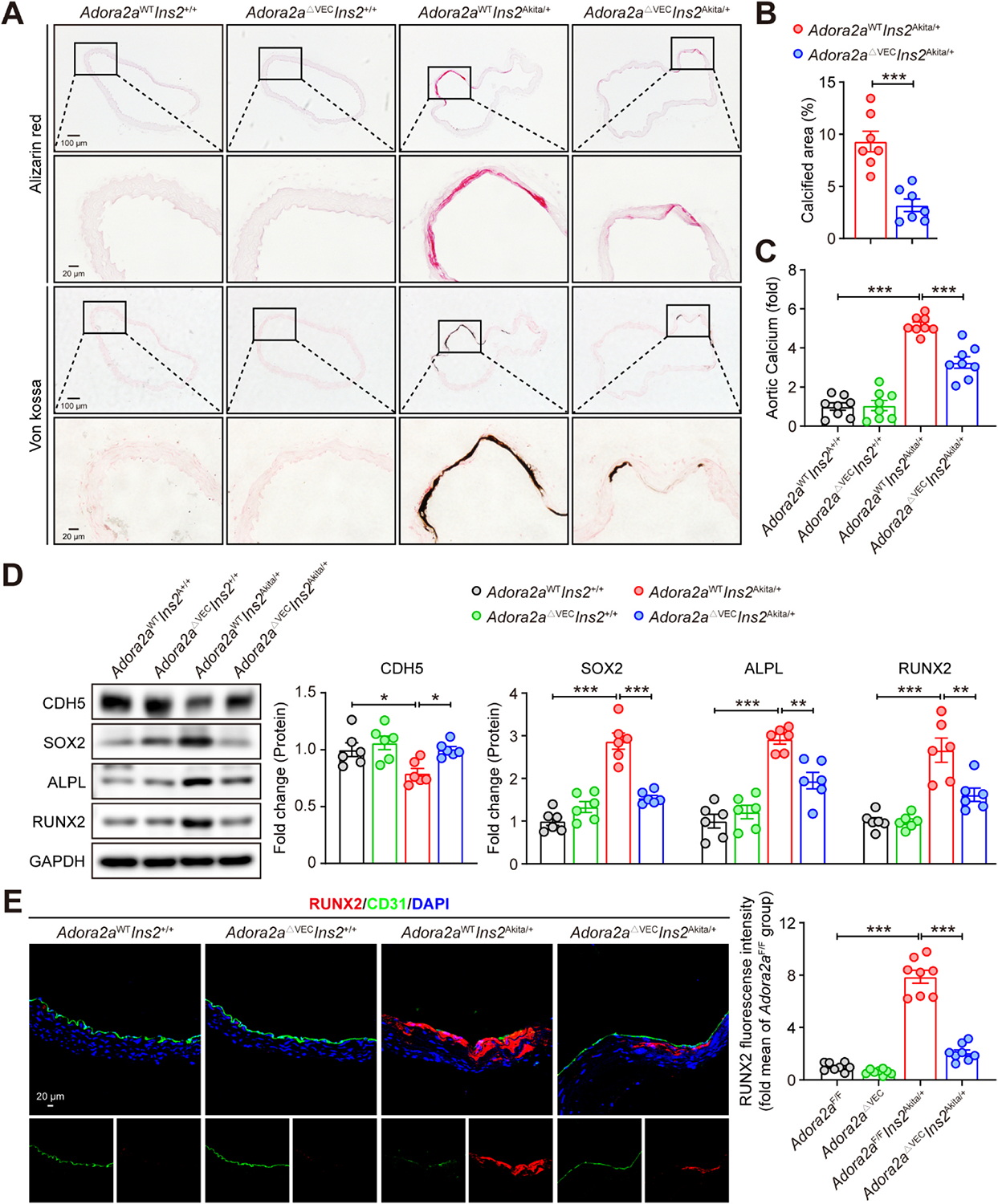
Endothelial *Adora2a* deficiency ameliorates aortic calcification in *Ins2*^Akita/+^ mice. **A-B**, Representative images of alizarin red- and von Kossa-stained aortic sections and quantification data of *Adora2a*^△VEC^ and *Ins2*^Akita/+^ mice (n = 7 mice per group). **C**, Total calcium content in the descending aortas of *Adora2a*^△VEC^ and *Ins2*^Akita/+^ mice (n = 8 mice per group). The results shown are normalized by dry weight. **D**, Western blot analysis and quantification data of protein levels of endothelial marker (CDH5), multipotent marker (SOX2), and osteogenic markers (ALPL and RUNX2) in thoracic aortas of *Adora2a*^△VEC^ and *Ins2*^Akita/+^ mice (n = 6 mice per group). **E**, Representative images of immunofluorescence staining and quantification data of RUNX2 (red) levels in thoracic aorta sections of *Adora2a*^△VEC^ and *Ins2*^Akita/+^ mice (n = 8 mice per group). Endothelial cells were marked with CD31 (green). The nucleus (blue) was stained with DAPI. The scale bar is 20 μm. Data are represented as means ± SEM. Statistical significance was determined by unpaired Student’s *t*-test (B), one-way ANOVA with the Bonferroni’s *post hoc* test (C, D), and Brown-Forsythe and Welch’s ANOVA tests with Dunnett’s T3 multiple comparison test (E). *p < 0.05, **p < 0.01, and ***p < 0.001 for indicated comparisons.

We next performed mineral staining to examine the extent of aortic calcification. Strikingly, aortic calcified areas in *Adora2a*^△VEC^*Ins2*^Akita/+^ mice were much less than that of *Adora2a*^WT^ *Ins2*^Akita/+^ mice (Figs. 2A and 2B). These results were further confirmed by calcium content determination with samples of aortic arteries (Fig. 2C). Then, we analyzed the effect of ADORA2A on osteogenic differentiation. Western blot assay suggested that endothelial *Adora2a* deficiency rescued the expression of endothelial marker vascular endothelial (VE)-cadherin (CDH5) and reduced the expression of SOX2, alkaline phosphatase (ALPL) and RUNX2 in *Ins2*^Akita/+^ aortas (Fig. 2D). Additionally, immunofluorescence staining showed that endothelial *Adora2a* deficiency greatly prevented the upregulation of RUNX2 in diabetic aortas, which came with reciprocal changes in the CD31 levels (Fig. 2E). These data demonstrate that limiting ADORA2A in endothelium decreases osteogenesis and calcification in diabetic aortas.

In addition, we also explored whether ADORA2A in VECs has a role in CKD-induced VC. *Adora2a*^ΔVEC^ mice and their WT control mice underwent 5/6th partial nephrectomy and followed a high phosphate diet and vitamin D supplementation to establish CKD-induced VC model (Supplementary Figure 4A). The histological analysis of the aortic wall was performed using hematoxylin and eosin (HE) staining. In the sham group, the aortas exhibited a consistent wall thickness and intact elastin fiber structure across the entire vessel. However, in the CKD group, the elastin fibers were disrupted. Notably, the structural abnormalities of the vascular wall in CKD mice were alleviated in *Adora2a*^ΔVEC^ mice when compared to the *Adora2a*^WT^ mice (Supplementary Figure 4B). Staining with alizarin red and von Kossa, along with calcium content determination of aortas, showed decreased calcification in aortas of *Adora2a*^ΔVEC^ mice when compared to the *Adora2a*^WT^ mice (Supplementary Figure 4B and 4C). Additionally, the effect of ADORA2A on osteogenic differentiation was evaluated through western blotting analysis. Along with the elevated aortic calcification, CKD mice exhibited upregulated osteogenic markers expression in the aortas compared to the sham-operated controls; however, this upregulation was inhibited in *Adora2a*^ΔVEC^ mice (Supplementary Figure 4D). Therefore, VEC-specific *Adora2a* deficiency exhibited a significant reduction of VC in CKD mice. Hence, this both adds to the evidence and helps confirm that ADORA2A in VECs is involved in developing VC.

### 3.3. ADORA2A is required for EndMT in cultured HAECs under high glucose treatment

**Fig. 3.**
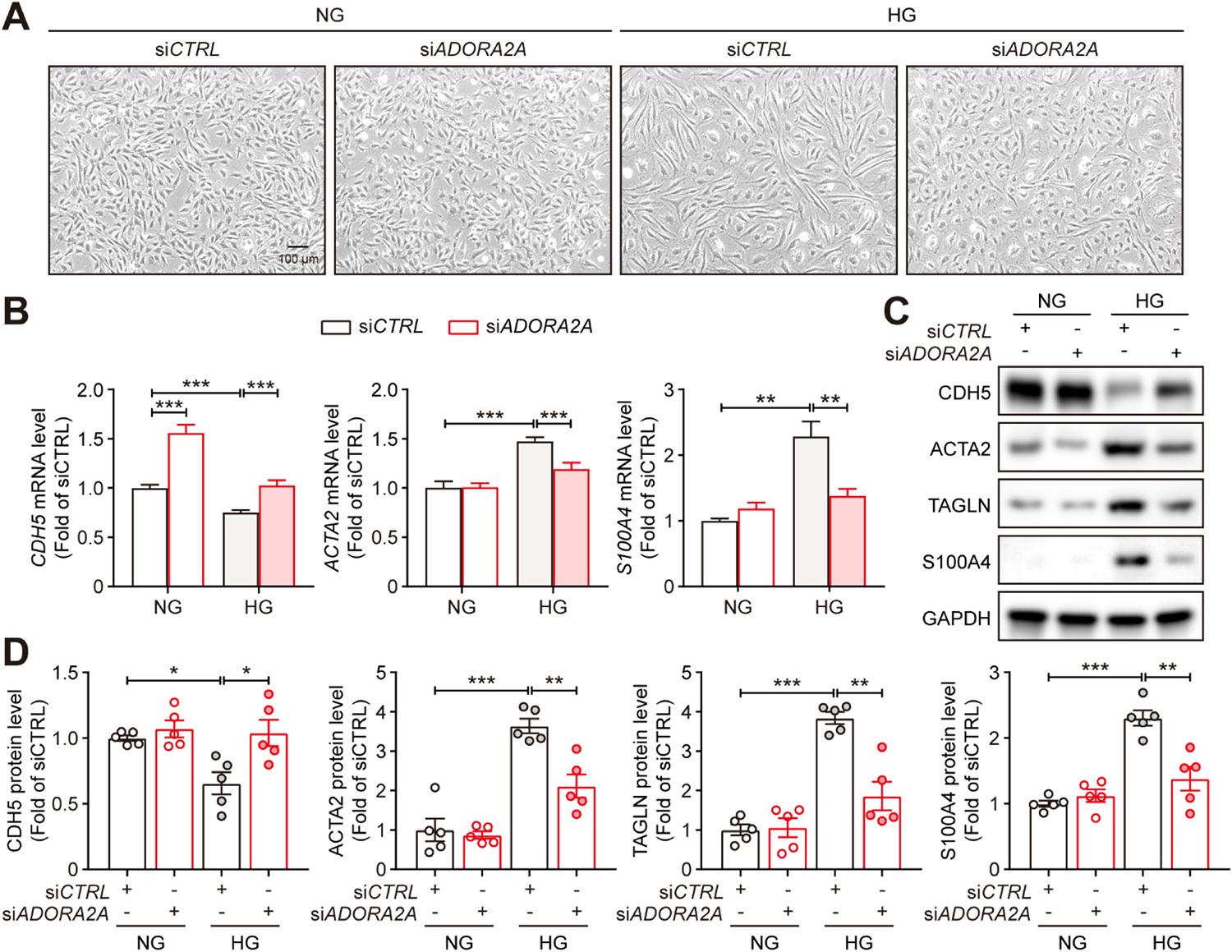
ADORA2A is required for EndMT in cultured HAECs with HG treatment. **A**, Representative images for cell morphological changes of HAECs exposed to mannitol (NG, 5.5 mM glucose and 19.5 mM mannitol) or high glucose (HG, 25 mM) for 72 h (n = 5). HAECs were transfected with si*CTRL* or si*ADORA2A*. **B**, Real-time PCR analysis of mRNA expression for endothelial marker (*CDH5*) and mesenchymal markers (*ACTA2* and *S100A4*) in HAECs exposed to mannitol (NG, 5.5 mM glucose and 19.5 mM mannitol) or high glucose (HG, 25 mM) for 48 h (n = 6). HAECs were transfected with si*CTRL* or si*ADORA2A*. **C-D**, Western blot analysis and quantification data of indicated protein levels for endothelial marker (CDH5) and mesenchymal markers (ACTA2, TAGLN and S100A4) in HAECs exposed to mannitol (NG, 5.5 mM glucose and 19.5 mM mannitol) or high glucose (HG, 25 mM) for 72 h (n = 5). HAECs were transfected with si*CTRL* or si*ADORA2A*. Data are represented as means ± SEM. Statistical significance was determined by one-way ANOVA followed by Bonferroni’s *post hoc* test (B, *CDH5* and *ACTA2*; D), and Brown-Forsythe and Welch’s ANOVA tests with Dunnett’s T3 multiple comparison test (B, *S100A4*). *p < 0.05, **p < 0.01, and ***p < 0.001 for indicated comparisons.

To visualize EndMT under high glucose, cell morphology was recorded with bright field imaging. As shown in Fig. 3A, endothelial monolayers showed a typical cobblestone morphology under normal conditions; after being exposed to high glucose, HAECs lost their characteristic morphology and became fibroblast-like and spindle-shaped. However, we confirmed the knockdown efficiency of ADORA2A using siRNA in HAECs at both the mRNA and protein levels and found that the knockdown of *ADORA2A* partially reversed these changes (Fig. 3A, Supplementary Figure 5A and 5B). Then, we examined the expression of EndMT-associated markers. The protein and mRNA levels of endothelial marker CDH5 were decreased while the mesenchymal markers, including alpha-smooth muscle actin (ACTA2), transgelin/smooth muscle 22 alpha (TAGLN, also known as SM22a), and S100 calcium binding protein A4/fibroblast-specific protein 1 (S100A4, also known as FSP1), were increased in HAECs exposed to high glucose, which were inhibited by *ADORA2A* knockdown (Figs. 3B, 3C and 3D). Together, these results indicate that ADORA2A is an important regulator of high glucose-induced EndMT.

### 3.4. ADORA2A knockdown inhibits EndMT and osteogenesis in HAECs under pro-calcified conditions

**Fig. 4.**
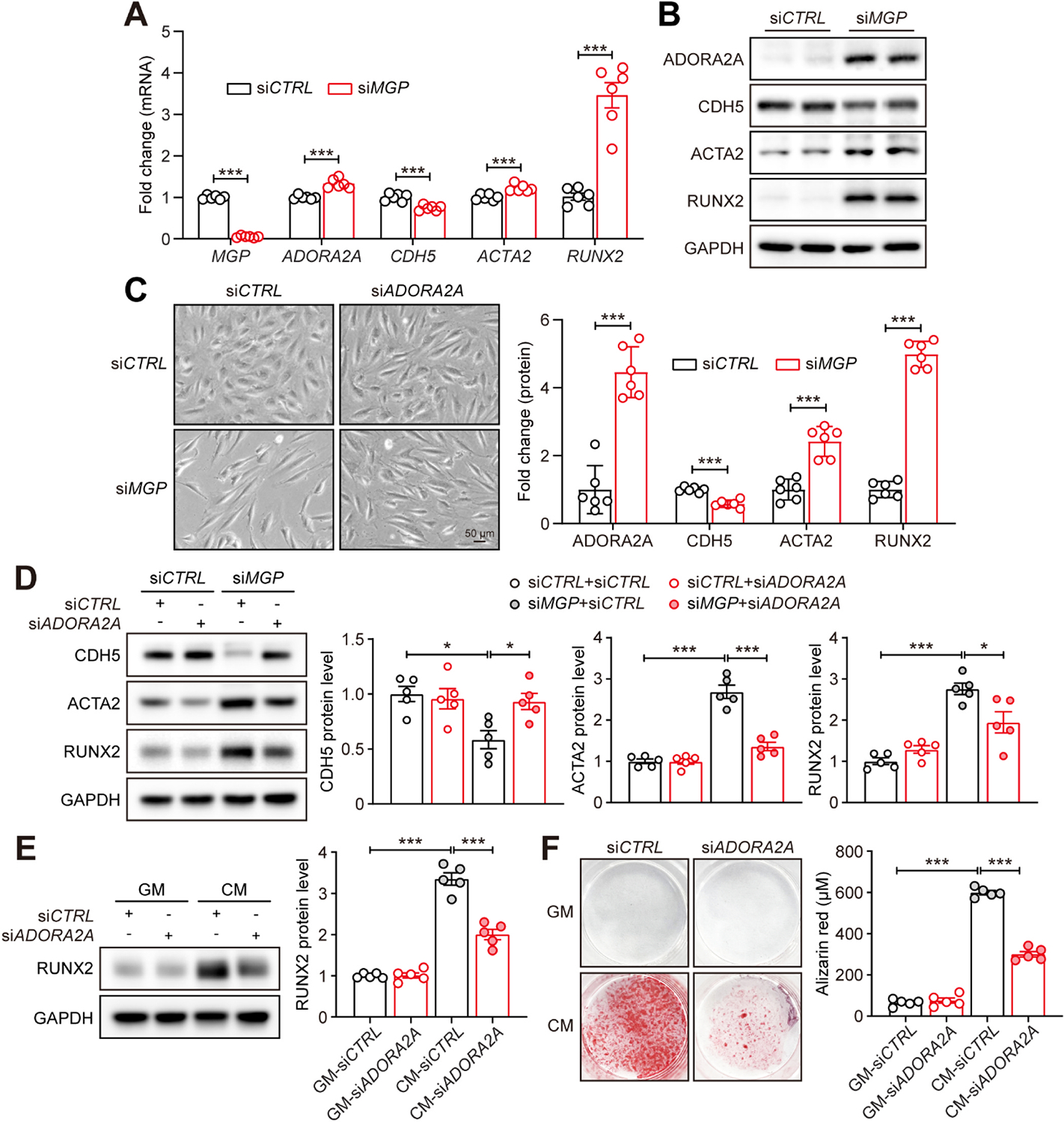
*ADORA2A* knockdown inhibits EndMT and osteogenesis in HAECs under pro-calcified conditions. **A**, Real-time PCR analysis of mRNA expression for *MGP*, *ADORA2A*, endothelial marker (*CDH5*), mesenchymal marker (*ACTA2*), and osteogenic marker (*RUNX2*) in *MGP*-depleted HAECs for 24 h (n = 6). HAECs were transfected with si*CTRL* or si*MGP*. **B**, Western blot analysis and quantification data of indicated protein levels for ADORA2A, endothelial marker (CDH5), mesenchymal marker (ACTA2) and osteogenic marker (RUNX2) in *MGP*-depleted HAECs for 48 h (n = 6). HAECs were transfected with si*CTRL* or si*MGP*. **C**, Representative images for cell morphological changes of *MGP* knockdown HAECs (n = 6). HAECs were co-transfected with si*CTRL*, si*MGP* and/or si*ADORA2A* for 48 h. **D**, Western blot analysis and quantification data of indicated protein levels for endothelial marker (CDH5), mesenchymal marker (ACTA2), and osteogenic marker (RUNX2) in *MGP* knockdown HAECs (n = 5). HAECs were co-transfected with si*CTRL*, si*MGP* and/or si*ADORA2A* for 48 h. **E**, Western blot analysis and quantification data of indicated protein levels for osteogenic marker (RUNX2) in HAECs exposed to GM or CM for 7 days (n = 5). HAECs were transfected with si*CTRL* or si*ADORA2A*. **F**, Representative images and quantification of alizarin red-stained HAECs exposed to GM or CM for 7 days (n = 5). HAECs were transfected with si*CTRL* or si*ADORA2A*. Data are represented as means ± SEM. Statistical significance was determined by unpaired Student’s *t*-test (A, C) and one-way ANOVA followed by Bonferroni’s *post hoc* test (D, E, and F). *p < 0.05, and ***p < 0.001 for indicated comparisons.

To further clarify the regulatory role of ADORA2A in EndMT underlying VC, we established *in vitro* models of EC calcification. Matrix GLA protein (MGP), a vitamin K-dependent protein, has been confirmed as an important inhibitor of VC. We knocked down *MGP* in HAECs by siRNA transfection to induce EndMT *in vitro*. Transduction of si*MGP* reduced the mRNA transcript level of *MGP* by over 90 % (Fig. 4A). As a result, the knockdown of *MGP* led to decreased expression of endothelial marker CDH5 and increased expression of mesenchymal markers ACTA2 and osteogenic marker RUNX2, suggesting that the EndMT and osteogenesis occurred (Figs. 4A and 4B). Of note, ADORA2A expression in *MGP*-depleted HAECs was significantly increased at both mRNA and protein levels (Figs. 4A and 4B). To examine the participation of ADORA2A in EndMT induction, si*MGP* and si*ADORA2A* were co-transfected to HAECs. As depicted in Fig. 4C, HAECs transfected with si*CTRL* displayed a classic cobble-stone pattern. In contrast, cells transfected with si*MGP* lost their cellular features and evolved into a spindle-shaped elongated cell morphology. *ADORA2A* knockdown with si*ADORA2A* restored the expression of EndMT-associated markers (CDH5 and ACTA2) and osteogenic marker RUNX2 in *MGP* knockdown HAECs (Fig. 4D). Moreover, we treated the cells with CM to induce calcium deposits, whereas GM was used as the control group. *ADORA2A* knockdown significantly decreased the expression of osteogenic marker RUNX2 (Fig. 4E), and calcium nodule formation, as shown by alizarin red staining (Fig. 4F). These results indicate that ADORA2A is involved in the induction of EndMT and subsequent calcium deposition.

### 3.5. ADORA2A overexpression aggravates EndMT and osteogenesis in HAECs

**Fig. 5.**
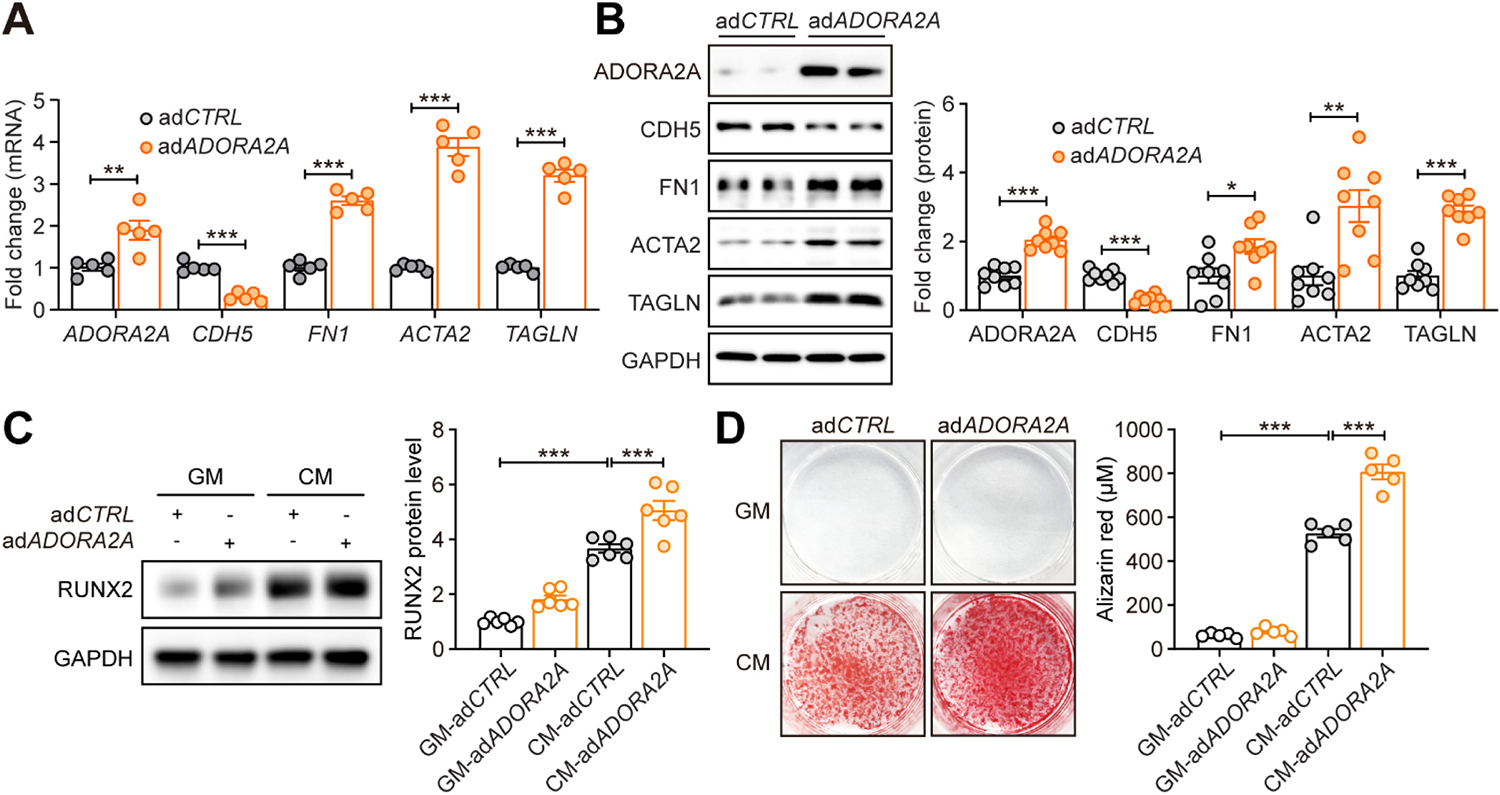
*ADORA2A* overexpression aggravates EndMT and osteogenesis in HAECs. **A**, Real-time PCR analysis of mRNA expression for *ADORA2A*, endothelial marker (*CDH5*), and mesenchymal marker (*FN1*, *ACTA2* and *TAGLN*) in ad*CTRL*- or ad*ADORA2A*-infected HAECs for 3 days (n = 6). **B**, Western blot analysis and quantification data of indicated protein levels for ADORA2A, endothelial marker (CDH5) and mesenchymal marker (FN1, ACTA2 and TAGLN) in ad*CTRL*- or ad*ADORA2A*-infected HAECs for 5 days (n = 8). **C**, Western blot analysis and quantification data of indicated protein levels for osteogenic markers (RUNX2) in HAECs exposed to GM or CM for 7 days (n = 6). HAECs were infected with ad*CTRL* or ad*ADORA2A*. **D**, Representative images and quantification of alizarin red-stained HAECs exposed to GM or CM for 7 days (n = 5). HAECs were infected with ad*CTRL* or ad*ADORA2A*. Data are represented as means ± SEM. Statistical significance was determined by unpaired Student’s *t*-test (A, B) and one-way ANOVA followed by Bonferroni’s *post hoc* test (C, D). *p < 0.05, **p < 0.01, and ***p < 0.001 for indicated comparisons.

In addition to the above loss-of-function studies, gain-of-function experiments with VECs overexpressing *ADORA2A* were also performed. We carried out ADORA2A gain-of-function studies using adenoviral vectors. The mRNA and protein levels of endothelial marker CDH5 decreased in HAECs with overexpression of *ADORA2A* (Figs. 5A and 5B). These *ADORA2A* overexpressing cells also exhibited the increased expression of EndMT-associated markers, including fibronectin 1 (FN1), ACTA2 and TAGLN (Figs. 5A and 5B). Additionally, we treated HAECs with CM to induce osteogenesis. *ADORA2A* overexpression significantly increased the expression of osteogenic marker RUNX2 (Fig. 5C), and calcium deposition quantified by alizarin red staining (Fig. 5D). Altogether, these data suggest that *ADORA2A* overexpression aggravates EndMT and osteogenesis of HAECs *in vitro*.

### 3.6. Pharmacological inhibition of ADORA2A protects against diabetic vascular calcification

**Fig. 6.**
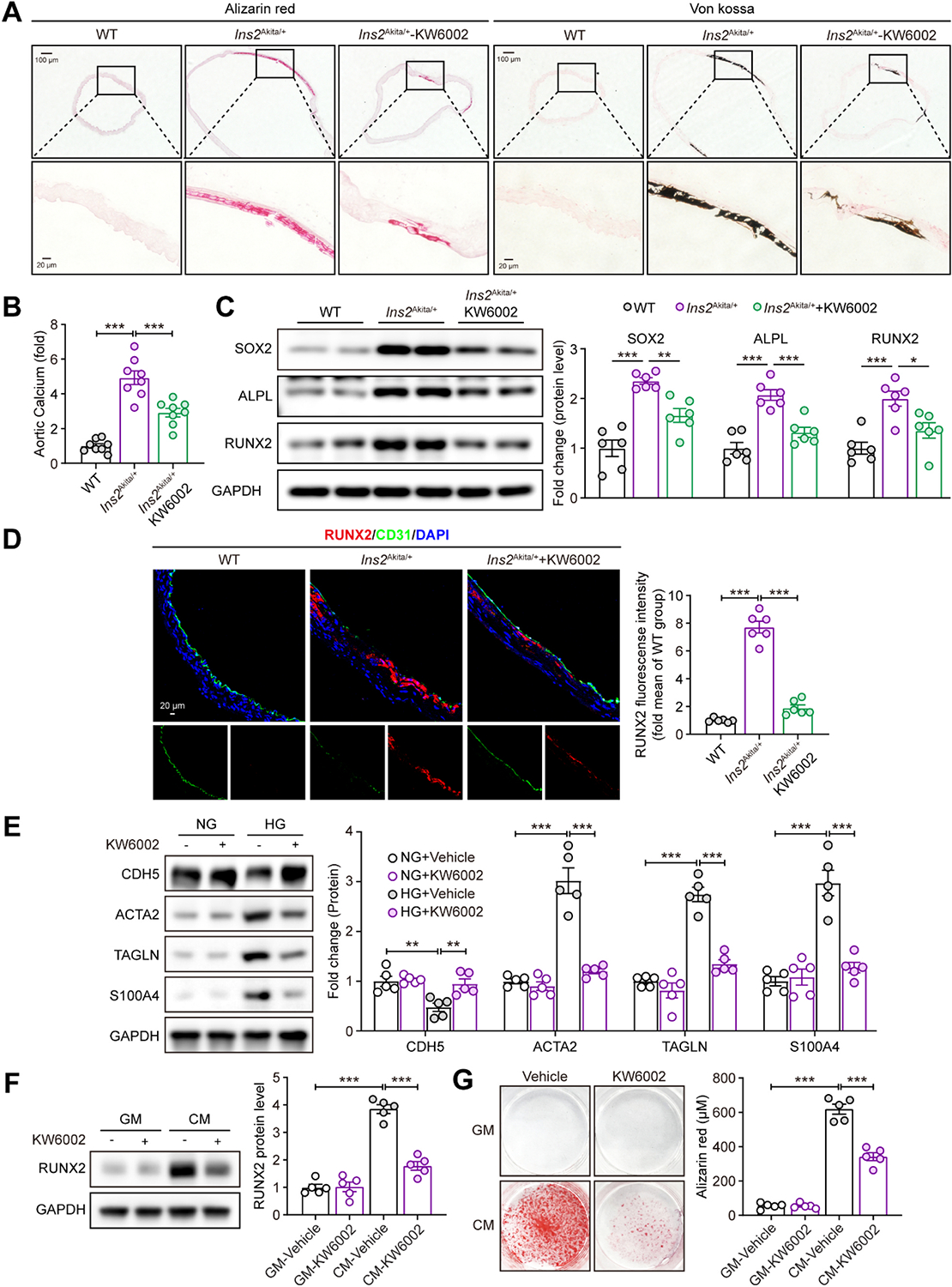
Pharmacological inhibition of ADORA2A protects against diabetic vascular calcification. **A**, Representative images of alizarin red- and von Kossa-stained aortic sections of vehicle- and KW6002-treated *Ins2*^Akita/+^ mice (n = 8 mice per group). **B**, Total calcium content in the descending aortas of vehicle- and KW6002-treated *Ins2*^Akita/+^ mice (n = 8 mice per group). The results shown are normalized by dry weight. **C**, Western blot analysis and quantification of indicated protein expression for multipotent marker (SOX2) and osteogenic markers (ALPL and RUNX2) in thoracic aortas of vehicle- and KW6002-treated *Ins2*^Akita/+^ mice (n = 6 mice per group). **D**, Representative images of immunofluorescence staining and quantification data of RUNX2 (red) levels in thoracic aorta sections of vehicle-and KW6002-treated *Ins2*^Akita/+^ mice (n = 6 mice per group). Endothelial cells were marked with CD31 (green). Nucleus (blue) was stained with DAPI. The scale bar is 20 μm. **E**, Western blot analysis and quantification of indicated protein expression for endothelial marker (CDH5) and mesenchymal marker (ACTA2, TAGLN and S100A4) in vehicle- or KW6002-treated HAECs exposed to mannitol (NG, 5.5 mM glucose and 19.5 mM mannitol) or high glucose (HG, 25 mM) for 72 h (n = 5). **F**, Western blot analysis and quantification of indicated protein expression for osteogenic markers (RUNX2) in vehicle- or KW6002-treated HAECs exposed to GM or CM for 7 days (n = 5). **G**, Representative images and quantification of alizarin red-stained HAECs with vehicle or KW6002 treatment exposed to GM or CM for 7 days (n = 5). Data are represented as means ± SEM. Statistical significance was determined by one-way ANOVA followed by Bonferroni’s *post hoc* test (B, C, E, F, and G), and Brown-Forsythe and Welch’s ANOVA tests with Dunnett’s T3 multiple comparison test (D). *p < 0.05, **p < 0.01, and ***p < 0.001 for indicated comparisons.

Besides genetically engineered mice, we also used an ADORA2A antagonist KW6002, to test whether pharmacological inhibition of ADORA2A protects against VC in diabetic mice. As expected, the treatment with KW6002 effectively mitigated calcified areas (Fig. 6A), calcium content (Fig. 6B), and osteogenic differentiation (Figs. 6C and 6D) in diabetic mice. In addition, in the *in vitro* assay, KW6002 treatment restrained the loss of endothelial marker CDH5 and the upregulation of mesenchymal markers (ACTA2 and S100A4) and osteogenic marker RUNX2 in HAECs induced by high glucose (Fig. 6E). Moreover, *MGP* knockdown-induced EndMT and osteogenesis activation was also significantly abolished with KW6002 treatment (Supplementary Figure 6). Under osteogenic medium induction, the expression of osteogenic marker RUNX2 was inhibited by KW6002 treatment (Fig. 6F). Along with this, mineral staining also revealed that KW6002 treatment substantially limited calcified nodule formation in HAECs after 14 days (Fig. 6G). Altogether, these findings indicate that pharmacological inhibition of ADORA2A attenuates aortic osteogenesis and calcium accumulation under diabetic conditions.

### 3.7. CREB1 and SNAI1 are involved in ADORA2A-mediated EndMT

**Fig. 7.**
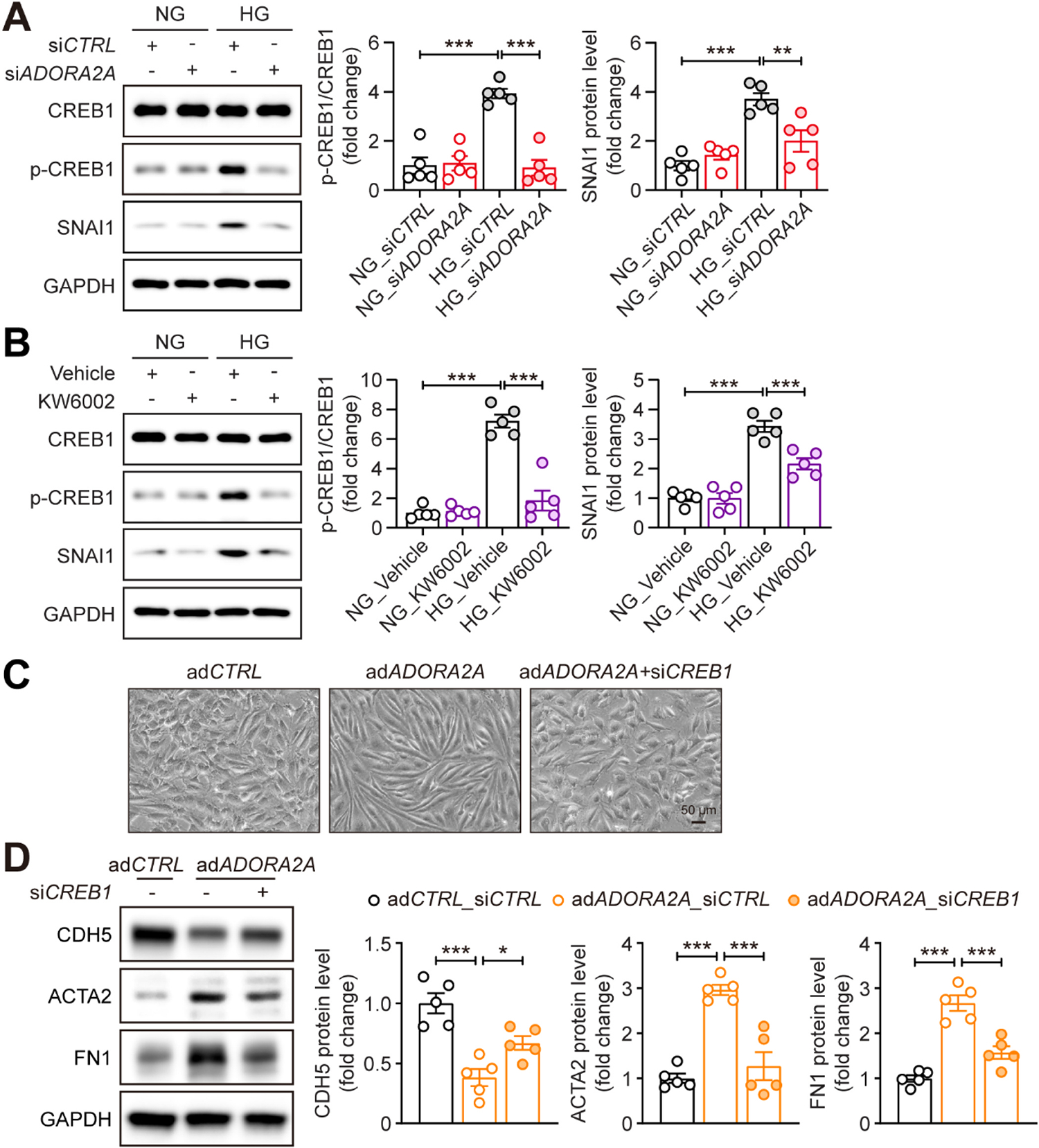
CREB1 and SNAI1 are involved in ADORA2A-mediated EndMT. **A**, Western blot analysis and quantification of indicated protein expression for CREB1, p-CREB1 and SNAI1 in HAECs exposed to mannitol (NG, 5.5 mM glucose and 19.5 mM mannitol) or high glucose (HG, 25 mM) for 72 h (n = 5). HAECs were transfected with si*CTRL* or si*ADORA2A*. **B**, Western blot analysis and quantification of indicated protein expression for CREB1, p-CREB1 and SNAI1 in vehicle- or KW6002-treated HAECs exposed to mannitol (NG, 5.5 mM glucose and 19.5 mM mannitol) or high glucose (HG, 25 mM) for 72 h (n = 5). **C**, Representative images for cell morphological changes of ad*CTRL*- or ad*ADORA2A*-infected HAECs for 5 days (n = 5). HAECs were transfected with si*CTRL* or si*CREB1*. **D**, Western blot analysis and quantification of indicated protein expression for endothelial marker (CDH5) and mesenchymal marker (ACTA2 and FN1) in ad*CTRL*- or ad*ADORA2A*-infected HAECs for 5 days (n = 5). HAECs were transfected with si*CTRL* or si*CREB1*. Data are represented as means ± SEM. Statistical significance was determined by one-way ANOVA followed by Bonferroni’s *post hoc* test. *p < 0.05, **p < 0.01, and ***p < 0.001 for indicated comparisons.

As a typical GPCR, ADORA2A participates in many pathophysiological processes through cAMP/PKA/CREB1 signaling pathway. Of note, in the present study, we found that CREB1 was more prominently phosphorylated in HAECs treated with high glucose compared to cells treated with normal glucose and this increased CREB1 phosphorylation was attenuated by *ADORA2A* knockdown or KW6002 treatment (Figs. 7A and 7B). Meanwhile, the protein level of SNAI1 also increased in HAECs under high glucose treatment, which was inhibited by *ADORA2A* knockdown or KW6002 treatment (Figs. 7A and 7B). Subsequently, gain-of-function assays with *ADORA2A* overexpression in HAECs were used further to examine the role of CREB1 in ADORA2A-mediated osteogenesis. We co-transfected si*CREB1* to knock down *CREB1* expression in VECs transfected with *ADORA2A* overexpression. As shown in Figs. 7C and 7D, *ADORA2A* overexpression induced HAECs to undergo EndMT as shown with changes of morphology and EndMT-associated markers (CDH5, ACTA2, and FN1); however, these changes caused by *ADORA2A* overexpression were explicitly reversed by *CREB1* knockdown with si*CREB1*, whose knockdown efficiency was validated at both the mRNA and protein levels (Supplementary Figure 5C and 5D). Collectively, these results suggest that CREB1 and SNAI1 are involved in ADORA2A-mediated EndMT, and CREB1 activation in VECs is critical for ADORA2A-mediated osteogenesis and VC.

### 3.8. ADORA2A recruits CREB1 onto SNAI1 promoter to trigger SNAI1 expression

**Fig. 8.**
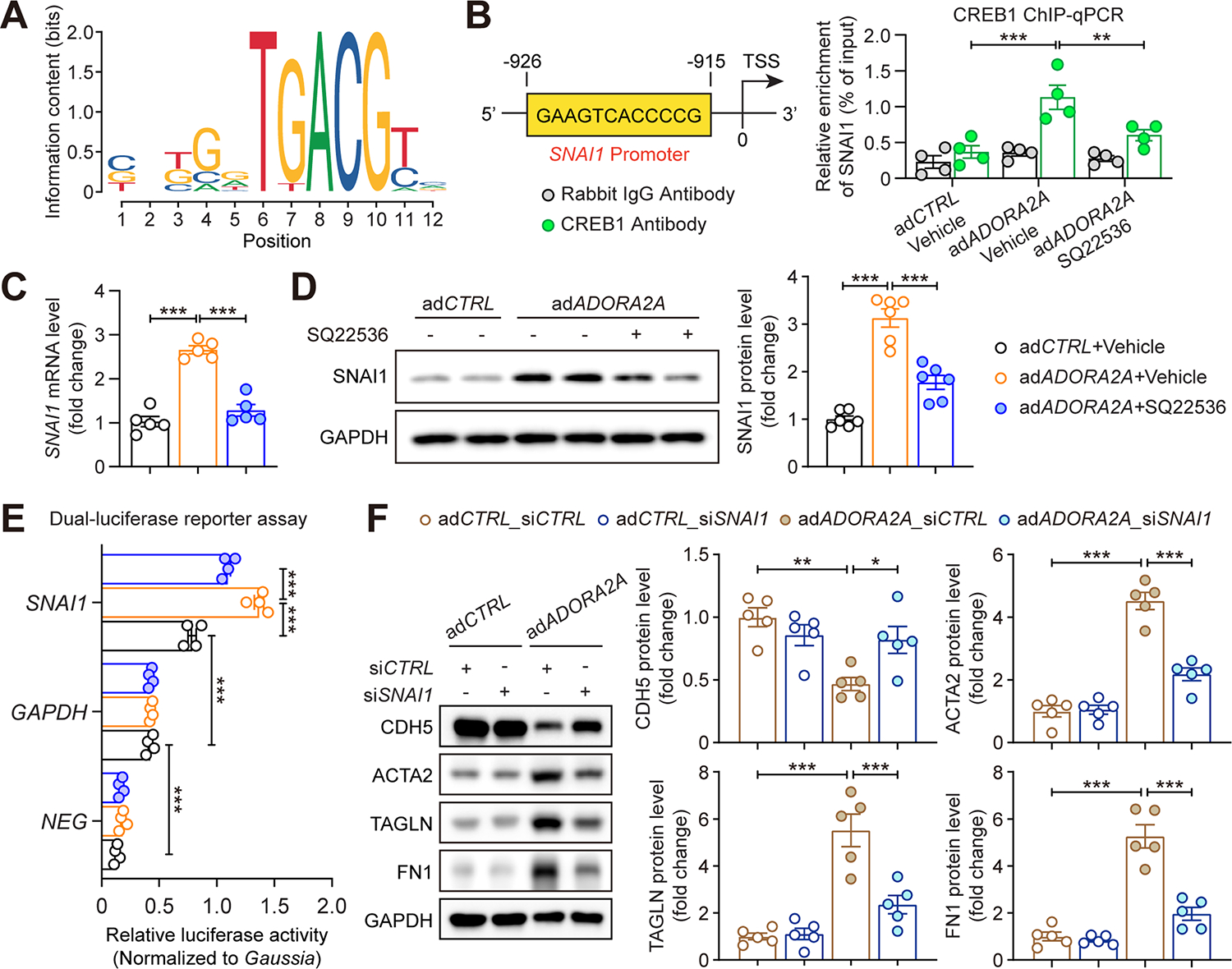
ADORA2A recruits CREB1 onto the *SNAI1* promoter to trigger *SNAI1* expression. **A**, Binding domain of CREB1 on the *SNAI1* promoter was predicted by JASPAR database. **B**, Binding domain of CREB1 on the *SNAI1* promoter was verified by ChIP-qPCR analysis in ad*CTRL*- or ad*ADORA2A*-infected HAECs treated with vehicle or SQ22536 (10μM) for 5 days (n = 4). **C**, Real-time PCR analysis of mRNA expression for *SNAI1* in ad*CTRL*- or ad*ADORA2A*-infected HAECs treated with vehicle or SQ22536 (10 μM) for 3 days (n = 5). **D**, Western blot analysis and quantification of SNAI1 protein expression in ad*CTRL*- or ad*ADORA2A*-infected HAECs treated with vehicle or SQ22536 (10 μM) for 5 days (n = 6). **E**, The *SNAI1* luciferase promoter activity in HAECs transfected with ad*CTRL* or ad*ADORA2A* and treated with SQ22536 (10 μM) for 5 days (n = 4). **F**, Western blot analysis and quantification of indicated protein expression for endothelial marker (CDH5) and mesenchymal marker (ACTA2, TAGLN and FN1) in ad*CTRL*- or ad*ADORA2A*-infected HAECs for 5 days (n = 5). HAECs were transfected with si*CTRL* or si*SNAIL1*. Data are represented as means ± SEM. Statistical significance was determined by one-way ANOVA followed by Bonferroni’s *post hoc* test (B, C, D, and F), and two-way ANOVA (E). *p < 0.05, **p < 0.01, and ***p < 0.001 for indicated comparisons.

As a regulatory transcription factor, SNAI1 has been reported to be involved in hypoxia-induced EndMT of human coronary endothelial cells [27]. To investigate whether the cAMP/PKA/CREB1 signaling pathway is involved in ADORA2A-mediated SNAI1 activation, we identified potential CREB1 binding sites within the *SNAI1* promoter using the JASPAR database (Fig. 8A). Subsequent ChIP-qPCR analysis confirmed the binding domain of CREB1 on the *SNAI1* promoter in VECs overexpressing *ADORA2A* (Fig. 8B). However, this interaction was effectively blocked by SQ22536, a specific adenylate cyclase inhibitor that reduces intracellular cAMP levels, leading to the inactivation of CREB1 (Fig. 8B). Consistently, alterations in mRNA and protein levels of SNAI1 in ad*CTRL*- or ad*ADORA2A*-infected HAECs corroborated these findings (Figs. 8C and 8D). We further assessed whether CREB1 could regulate the *SNAI1* promoter. Functionally, a GLuc-ON human *SNAI1* promoter reporter construct (−1225 to +68 relative to the transcription start site, cloned into the pEZX-PG04.1 vector) was employed, together with NEG and GAPDH control reporters. We found that *ADORA2A* overexpression markedly enhanced *SNAI1* promoter-driven *Gaussia* luciferase activity (normalized to the SEAP internal control) in HAECs compared with ad*CTRL*, whereas this induction was significantly abolished by SQ22536 (10 μM) treatment (Fig. 8E). Furthermore, the downregulation of endothelial marker CDH5 and upregulation of EndMT-associated markers (ACTA2, TAGLN, and FN1) induced by *ADORA2A* overexpression in HAECs were explicitly reversed by *SNAI1* knockdown with si*SNAI1* (Fig. 8F). In addition, the knockdown efficiency has been demonstrated in the Supplementary Figure 5E and 5F. Cumulatively, these results imply the requirement for the cAMP-CREB1-SNAI1 axis on ADORA2A-mediated EndMT and osteogenesis in VECs.

## 4. Discussion

In the current study, we showed that ADORA2A is highly expressed in the endothelium of diabetic mice as well as in cultured HAECs under high glucose treatment. Endothelial-specific depletion of *Adora2a* protects mice from diabetic VC through inhibiting EndMT and osteogenic differentiation. Moreover, the pharmacological inhibition of ADORA2A demonstrates the strong anti-calcification effects both *in vivo* and *in vitro*. Mechanistically, ADORA2A regulates the levels of CREB1 and SNAI1 in HAECs, which results in EndMT and subsequent VC progression. Taken together, our findings indicate that endothelial ADORA2A plays a critical role in the development of diabetic vascular complications.

The detrimental effects of DM on human health are predominantly due to its vascular complications, with VC being one of the major complications. DM patients have a significantly higher incidence of VC compared to non-DM patients [28]. However, the prevention and treatment of VC are not as straightforward as lowering blood glucose levels. Long-term intensive glycemic control may be detrimental to large vessel diseases and could not reduce the risk of death from cardiovascular events associated with vascular lesions [29]. Current therapeutic agents for VC include calcium channel blockers, vitamin K supplements, antioxidants, and statins. These drugs have certain therapeutic effects on VC, in particular, calcium channel blockers slowed arterial calcification in experimental animals. In humans, however, the experimental results of these drugs were not pronounced [30]. One possible reason is that most studies on VC have relied on animal models of renal insufficiency or calmodulin deletion, which may not fully represent human diseases. Thus, therapeutic interventions should be developed to specifically target diabetic VC with the aim of reducing cardiovascular events. In this study, *Ins2*^Akita/+^ mice were used to mimic human DM. Our findings revealed an upregulation of osteogenic markers and calcium deposition in the arteries of *Ins2*^Akita/+^ compared to the littermate non-diabetic control mice, consistent with observations in diabetic patients [31]. Combining with the characteristics of DM, studies on the pathogenesis of VC will contribute to a deeper understanding of the development and progression of DM and its cardiovascular complications. However, a limitation to the generalizability of the study is that it did not consider gender/sex issues.

ADORA2A is abundantly expressed in vascular cells [32], and genome-wide association studies (GWAS) have linked it to human vascular diseases [33]. ADORA2A-mediated endothelial injury has emerged as a critical factor in the pathogenesis of vascular injury diseases. Endothelial dysfunction initiates a cascade of events, including lipid deposition, inflammatory cell infiltration, and subsequent arterial disorders. Our previous studies revealed a substantial increase in ADORA2A expression within VECs in response to ischemic, hypoxic, and inflammatory stimuli; specific deletion of ADORA2A in VECs effectively attenuated the development of atherosclerosis and the severity of ischemic brain injury [25,34–36]. Consistently, inhibition of ADORA2A signaling by caffeine protected against oxygen-induced retinopathy in mice, further underscoring the role of ADORA2A in hypoxia-driven vascular injury [37]. Hyperglycemia and glucose fluctuations can severely impair endothelial function, which is the initiating event in VC. Here, in mice with DM, we observed a significant upregulation of ADORA2A in the arterial vessels, with a strong co-localization pattern with VEC marker CD31. Unignorably, DM pathophysiological processes such as hypoxia and inflammation may also contribute to ADORA2A upregulation. These findings imply that ADORA2A may be involved in regulating endothelial injury and calcification in diabetic vasculature. By employing mice with VEC-specific *Adora2a* deficiency, we systematically demonstrated that ADORA2A-mediated endothelial dysfunction is involved in the development of diabetic VC.

VC is a complex process orchestrated by the transformation of multiple cell types into osteoblast-like cells within the vascular microenvironment [8], including VSMCs, fibroblast cells, and VECs. The cellular origin of osteoblast-like cells remains an area of active investigation and considerable interest. Although VSMCs have been the primary focus of many studies, VECs are increasingly attracting attention with their osteogenic differentiation potential, which is why this study focuses on the effect of endothelial ADORA2A on VC development. In 2010, Medici *et al*. first confirmed that VECs can differentiate into osteoblasts and chondrocytes and express bone-like matrices, suggesting that VECs may be another source of osteoblast-like cells [38]. Subsequent studies have shown that inflammatory factors and bone morphogenetic proteins (BMPs) can promote EndMT, leading to calcification [39,40]. Notably, Yao *et al*. demonstrated in diabetic mice that VECs are the source of osteoblast-like cells in calcified vessels [12]. Consistent with these findings, our results show that high glucose induces EndMT in VECs and upregulates the expression of the osteogenic marker RUNX2. Furthermore, we found that knockdown of *ADORA2A* significantly inhibited high glucose-induced EndMT and RUNX2 overexpression, providing a mechanistic explanation for the reduced calcium deposition observed in aortas of *Adora2a*^△VEC^*Ins2*^Akita/+^ mice. The inhibition of EndMT is one of the major mechanisms underlying the therapeutic effect of ADORA2A inactivation in the development of diabetic VC.

CREB1 is a vital nuclear transcription factor that can be triggered by a variety of extracellular signals [41] and holds potential as a therapeutic target for cardiovascular diseases [42]. Different GPCRs, including ARs, are known to activate CREB1. Studies have shown that adenosine mainly acted on immune cells via ADORA2A and ADORA2B, which elevated intracellular cAMP levels and subsequently led to the phosphorylation of CREB1 [43]. Notably, ADORA2A enhanced ALK5 expression through the cAMP/PKA/CREB signaling pathway in aortic ECs, which activated TGFβ-Smad2/3 signaling and contributed to atherosclerotic EndMT [35]. However, whether this regulatory mechanism is implicated in diabetic EndMT has yet to be clarified. Our results demonstrated that ADORA2A modulated both phosphorylation of CREB1 and SNAI1 in VECs under high glucose-induced EndMT conditions. Moreover, CREB1 could bind to the promoter region of *SNAI1* to stimulate its transcription, and silencing *SNAI1* reversed the EndMT prompted by *ADORA2A* overexpression. These findings provide further evidence that the cAMP/PKA/CREB1 signaling pathway serves as a critical mediator in ADORA2A-driven EndMT.

Due to the complex pathogenesis and diverse cellular origins of diabetic VC, effective clinical treatments remain a significant challenge. However, the identification of risk factors and multiple regulatory pathways offers new avenues for intervention and therapy. Here, we found that the selective ADORA2A antagonist KW6002 significantly ameliorated aortic calcium deposition in diabetic mice, and this beneficial effect was achieved by inhibiting EndMT. KW6002, a caffeine-derived xanthine derivative, was approved in 2013 (Japan) and 2019 (U.S.) for the treatment of Parkinson’s disease (PD). As the first non-dopaminergic adjunctive therapy to levodopa, KW6002 represents a groundbreaking therapeutic agent for PD [44]. It should be noted that while the drug has known central nervous system (CNS)-related side effects in PD patients (such as dyskinesia, dizziness, and insomnia), the comprehensive safety profile established from thousands of clinical trials provides a unique advantage for its translational application. Moreover, the efficacy of monotherapy with ADORA2A antagonists has been demonstrated, suggesting potential benefits beyond combination therapy [45]. While repurposing a neurological drug demands careful consideration of long-term safety, the current absence of targeted therapies for diabetic vascular calcification exposes patients to significant cardiovascular mortality risk. In this context, the benefit of KW6002 in inhibiting a key pathological process may substantially outweigh the risk of its manageable side effects. While future clinical trial designs will need to focus on monitoring long-term safety, the existing data support its translational potential as a new treatment for diabetic VC. Targeting the ADORA2A with antagonists may open new avenues for treating various diseases. Based on our findings, ADORA2A antagonists hold promise as a novel class of drugs for the treatment of diabetic VC.

In conclusion, we demonstrated that ADORA2A expression is markedly upregulated in VECs under high glucose conditions, contributing to EndMT and osteogenic differentiation. Through both phenotypic characterization and mechanistic investigations, we showed that ADORA2A inactivation mitigates diabetic VC by modulating EndMT via the CREB1-SNAI1 signaling axis. Our findings provide novel insights into the pathogenesis of diabetic VC and underscore ADORA2A as a promising therapeutic target for managing VC in DM.

## Supplementary Material

1

## Data Availability

Data will be made available on request.

## Appendix A. Supporting information

Supplementary data associated with this article can be found in the online version at doi:10.1016/j.phrs.2025.107981.

## Nonstandard abbreviations and acronyms

CVD: cardiovascular disease
DM: diabetes mellitus
VC: vascular calcification
ARs: adenosine receptors
GPCRs: G protein-coupled receptors
BMPs: bone morphogenetic proteins
cAMP: cyclic adenosine monophosphate
ADORA2A: adenosine receptor 2 A
KW6002: istradefylline
EndMT: endothelial-mesenchymal transitions
VECs: vascular endothelial cells
HAECs: human aortic endothelial cells
CDH5: vascular endothelial (VE)-cadherin
FN1: fibronectin 1
ACTA2: alpha-smooth muscle actin
TAGLN: transgelin, also known as smooth muscle 22 alpha (SM22a)
S100A4: S100 calcium binding protein A4, also known as fibroblast-specific protein 1 (FSP1)
RUNX2: runt-related transcription factor 2
CREB1: cAMP response element-binding protein 1
SNAI1: snail family transcriptional repressor 1
