# Supplementary material for: Endothelial adenosine receptor 2A loss alleviates diabetic vascular calcification by blocking CREB1-SNAI1-driven EndMT
Source: Pharmacol Res. Author manuscript; Available in PMC 2026 Jul 3. (PMC13331242; doi:10.1016/j.phrs.2025.107981)
Supplement: 1 [file NIHMS2184295-supplement-1.docx]

**SUPPLEMENTAL MATERIALS**

**Endothelial adenosine receptor 2A loss alleviates diabetic vascular calcification by blocking CREB1-SNAI1-driven EndMT**

Yaqi Zhou^a, b, c, 1^, Dingwei Zhao^a, c, d, 1^, Qian Ma^c, d^, Jiean Xu^c^, Yongfeng Cai^c, d^, Qiuhua Yang^e^, Qingen Da^a^, Kian Sheridan^e^, Chunxiang Zhang^f^, Clint L. Miller^g, h^, Rajeev Malhotra^i^, Suowen Xu^j, k, l^, Mei Hong^a*^, and Yuqing Huo^c, d*^

**^1^These authors have contributed equally to this article.**

^a^State Key Laboratory of Chemical Oncogenomics, Key Laboratory of Chemical Genomics, School of Chemical Biology and Biotechnology, Peking University Shenzhen Graduate School, Shenzhen 518055, China

^b^Department of Physiology, Research Center of Basic Integrative Medicine, School of Basic Medical Sciences, Guangzhou University of Chinese Medicine, 232 Waihuan East Road, University Town, Guangzhou 510006, China

^c^Vascular Biology Center, Department of Cellular Biology and Anatomy, Medical College of Georgia, Augusta University, Augusta, GA 30912, USA

^d^Departments of Ophthalmology, Medicine and Molecular and Cellular Biology, Baylor College of Medicine, Houston, TX 77030, USA

^e^Department of Pharmacological Sciences, Stony Brook University, Stony Brook, NY 11794, USA

^f^Emory University, Atlanta, GA 30322, USA

^g^Department of Cardiology, Key Laboratory of Medical Electrophysiology, Ministry of Education, Institute of Cardiovascular Research, The Affiliated Hospital of Southwest Medical University, Southwest Medical University, Luzhou, 646000, China

^h^Department of Biochemistry and Molecular Genetics, University of Virginia, Charlottesville, VA 22908, USA

^i^Center for Public Health Genomics, University of Virginia, Charlottesville, VA 22908, USA

^j^Cardiovascular Research Center, Division of Cardiology, Department of Medicine, Massachusetts General Hospital, Harvard Medical School, Boston, MA 02114, USA

^k^Department of Endocrinology, Centre for Leading Medicine and Advanced Technologies of IHM, The First Affiliated Hospital of USTC, Division of Life Sciences and Medicine, University of Science and Technology of China, Hefei, 230001, China

^l^Anhui Provincial Key Laboratory of Metabolic Health and Panvascular Diseases, Hefei, 230001, China

^m^Institute of Endocrine and Metabolic Diseases, University of Science and Technology of China, Hefei, 230001, China

^*^**Correspondence:**

Yuqing Huo, MD, PhD

Departments of Ophthalmology, Medicine, and Molecular and Cellular Biology, Baylor College of Medicine, Houston, TX 77030, USA

Phone: 713-798-1912 (Office)

Email: [yuqing.huo@bcm.edu](mailto:yuqing.huo@bcm.edu)

Mei Hong, Ph.D

School of Chemical Biology and Biotechnology, Peking University Shenzhen Graduate School, Shenzhen 518055, China

Phone: +86 755-2153-9221

Email: meihong.sz@pku.edu.cn

**
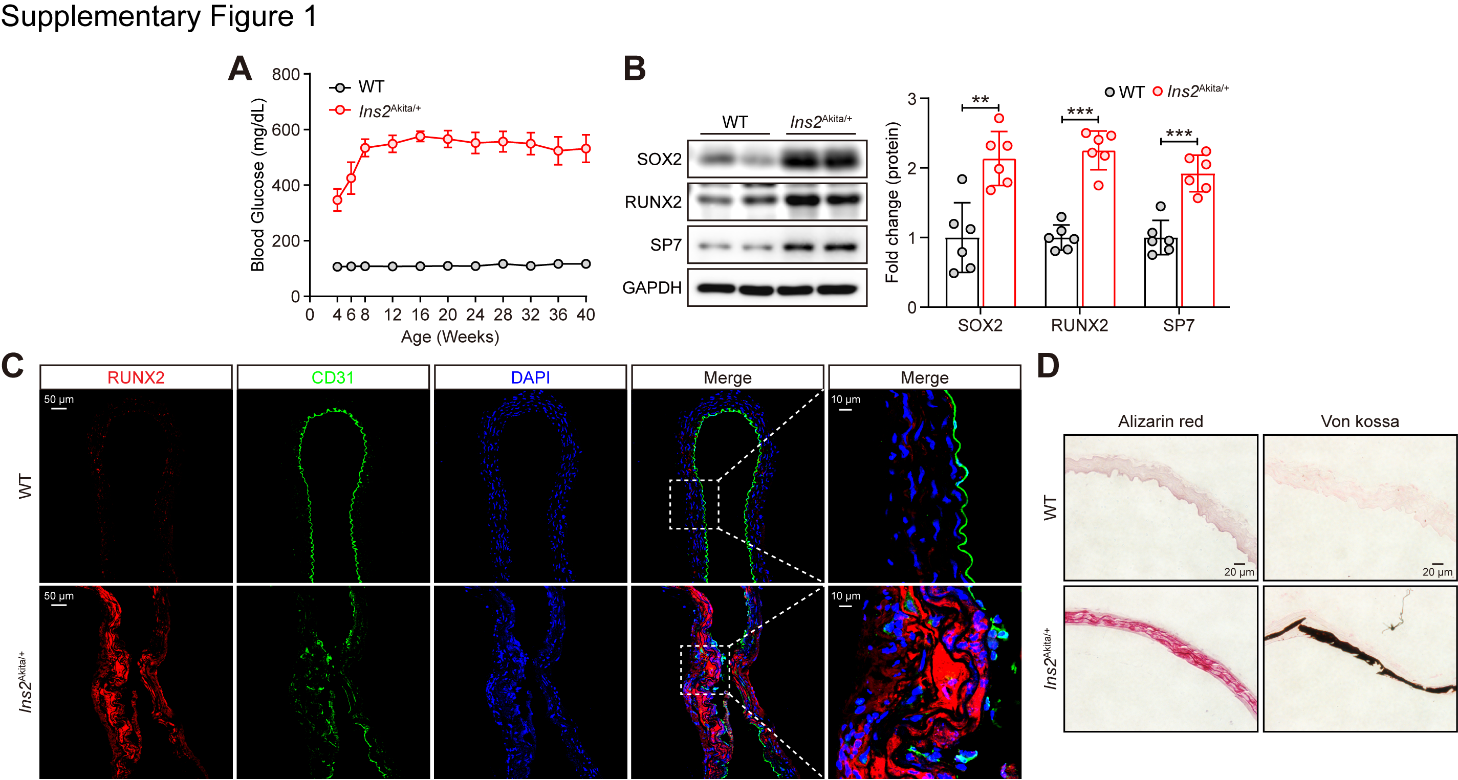
Supplemental Figures and Legends**

**Figure S1. Vascular calcification in *Ins2*^Akita/+^ mice.** **A**, Blood glucose levels in *Ins2*^Akita/+^ mice from 4 to 40 weeks of age (n = 6 mice per group). **B**, Western blot analysis and quantification of indicated protein expression for multipotent marker (SOX2) and osteogenic markers (RUNX2, SP7) in thoracic aortas of *Ins2*^Akita/+^ mice (n = 6 mice per group). **C**, Representative images of immunofluorescence staining and quantification data of RUNX2 (red) levels in thoracic aorta sections of *Ins2*^Akita/+^ mice (n = 5 mice per group). Endothelial cells were marked with CD31 (green). The nucleus (blue) was stained with DAPI. Scale bar is 10 or 50 μm. **D**, Representative images of alizarin red- and von Kossa-stained aortic sections of *Ins2*^Akita/+^ mice (n = 5 mice per group). Data are represented as means ± SEM. Statistical significance was determined by unpaired Student's *t*-test. ***p* < 0.01, and ****p* < 0.001 for indicated comparisons.

**
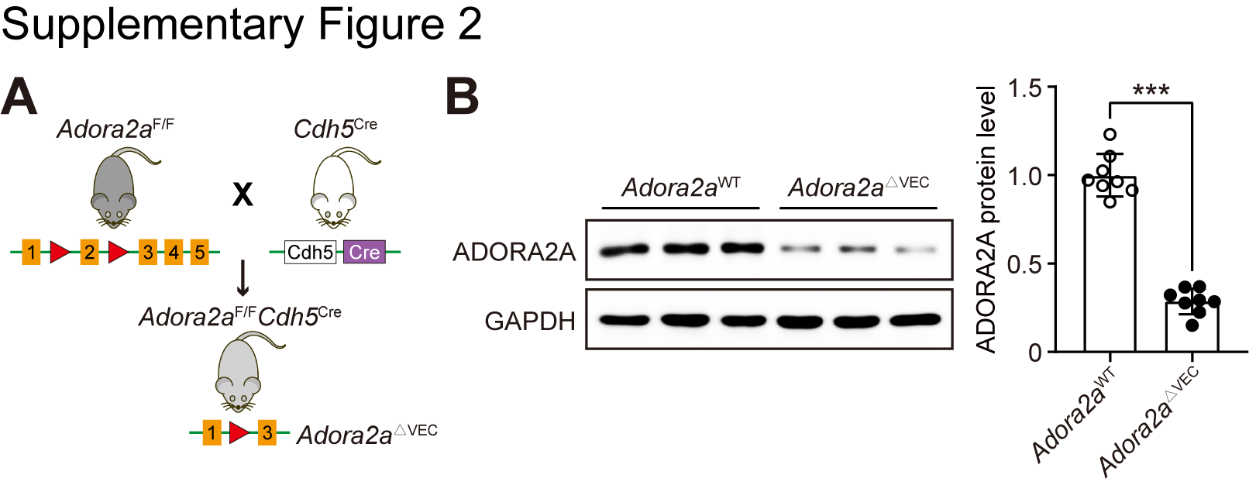
Figure S2. Generation of VEC-specific *Adora2a-deficient* mice.** **A**, Strategy for generating *Adora2a*^∆VEC^ mice by crossing *Adora2a*^F/F^ mice with *Cdh5*^Cre^ mice. **B**, Western blot analysis and quantification data of ADORA2A protein expression in VECs isolated from thoracic aortas of *Cdh5*^Cre^ (*Adora2a*^WT^) and *Adora2a*^∆VEC^ mice (n = 6 mice per group). Data are represented as means ± SEM. Statistical significance was determined by unpaired Student's *t*-test. ****p* < 0.001 for indicated comparisons.

**
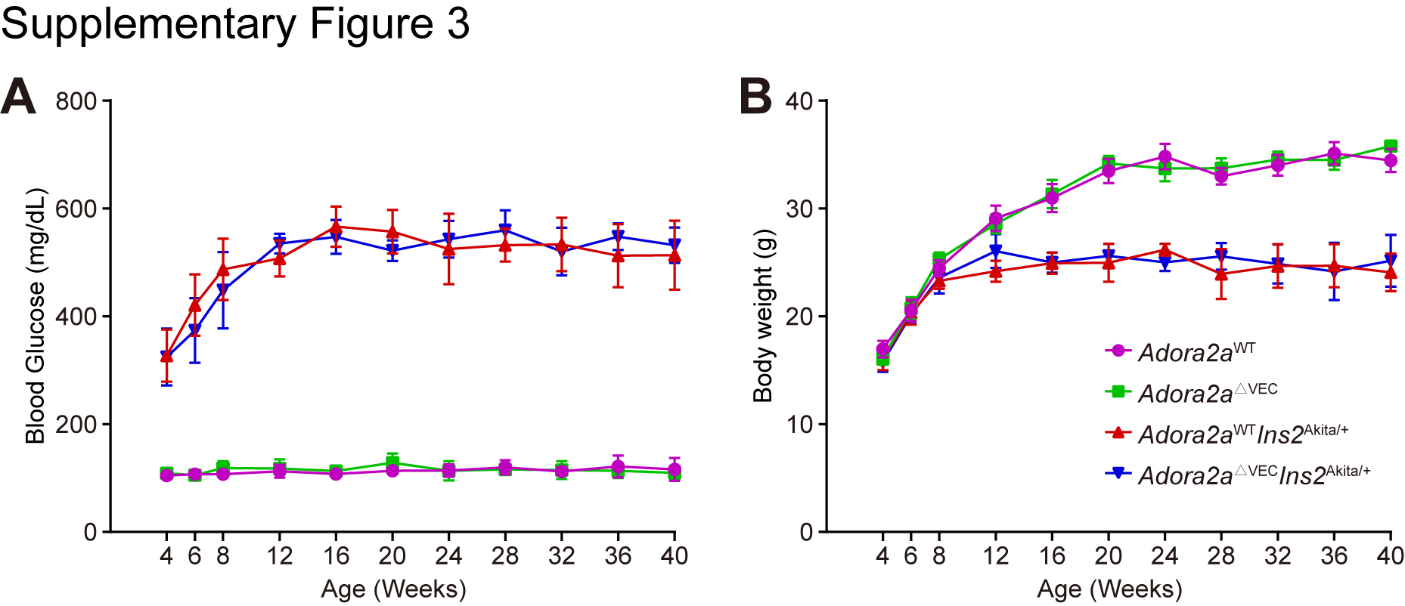
Figure S3. Characterization of blood glucose levels and body weights in *Adora2a*^△VEC^ and *Ins2*^Akita/+^ mice. A**, Blood glucose levels of *Adora2a*^△VEC^ and *Ins2*^Akita/+^ mice from 4 to 40 weeks of age (n = 6 mice per group). **B**, Body weight of *Adora2a*^△VEC^ and *Ins2*^Akita/+^ mice from 4 to 40 weeks of age (n = 6 mice per group). Data are represented as means ± SEM


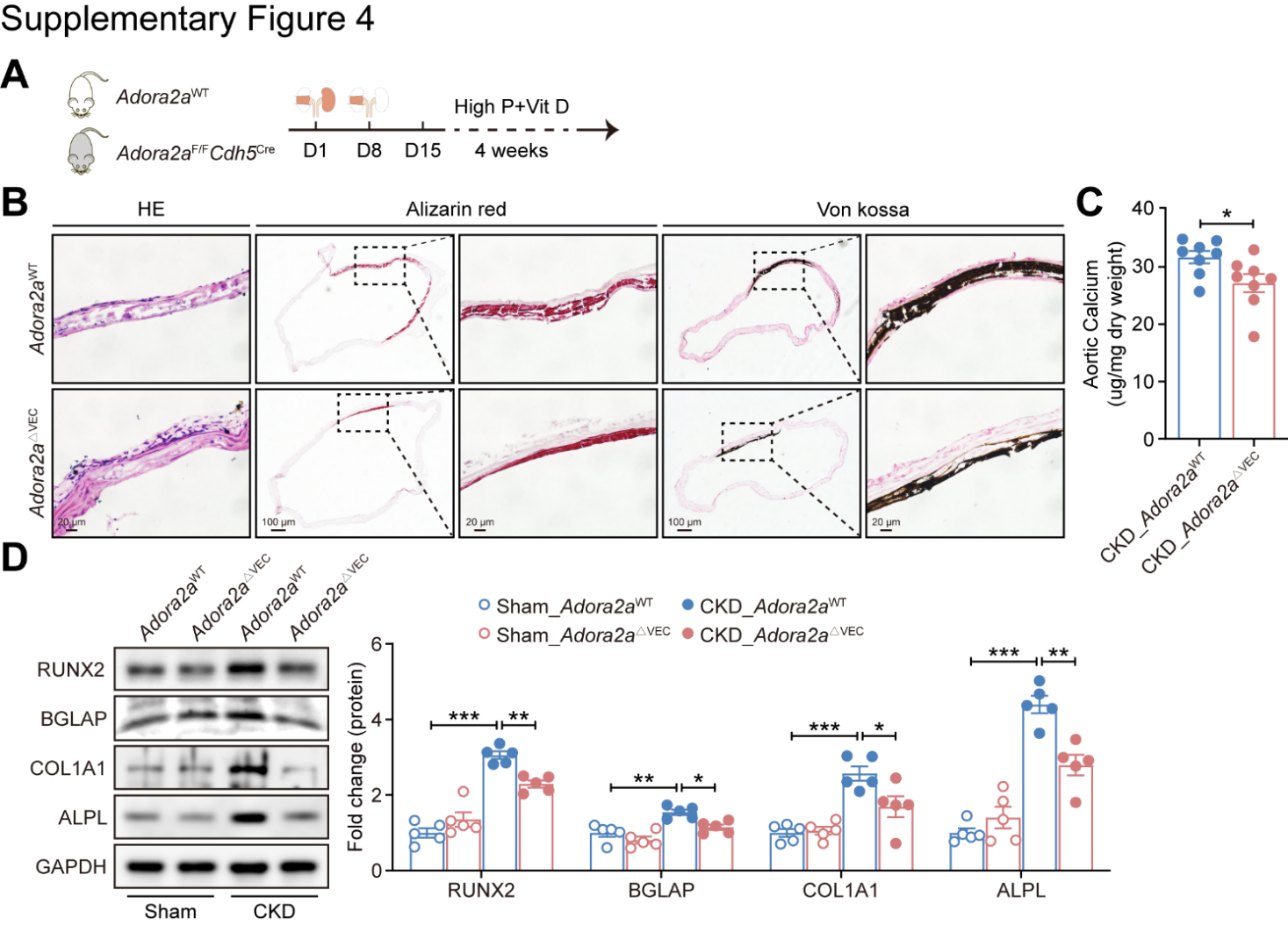
**Figure S4. VEC-specific *Adora2a* deficiency alleviates vascular calcification in CKD mice. A**, Schematic of the experimental design for CKD-associated vascular calcification model in *Cdh5*^Cre^ (*Adora2a*^WT^) and *Adora2a*^∆VEC^ mice. **B**, Representative images of HE-, alizarin red-, and von Kossa-stained thoracic aorta sections of CKD-associated vascular calcification model in *Cdh5*^Cre^ (*Adora2a*^WT^) and *Adora2a*^∆VEC^ mice (n = 8 mice per group). **C**, Total calcium content in the descending aortas of CKD *Cdh5*^Cre^ (*Adora2a*^WT^) and *Adora2a*^∆VEC^ mice (n = 8 mice per group). The results shown are normalized by dry weight. **D**, Western blot analysis and quantification of indicated protein expression for osteogenic markers (RUNX2, BGLAP, COL1A1, and ALPL) in thoracic aortas of CKD *Cdh5*^Cre^ (*Adora2a*^WT^) and *Adora2a*^∆VEC^ mice (n = 5 mice per group). Data are represented as means ± SEM. Statistical significance was determined by unpaired Student's *t*-test (C) and one-way ANOVA followed by Bonferroni’s *post hoc* test (D). **p* < 0.05, ***p* < 0.01, and ****p* < 0.001 for indicated comparisons.

**
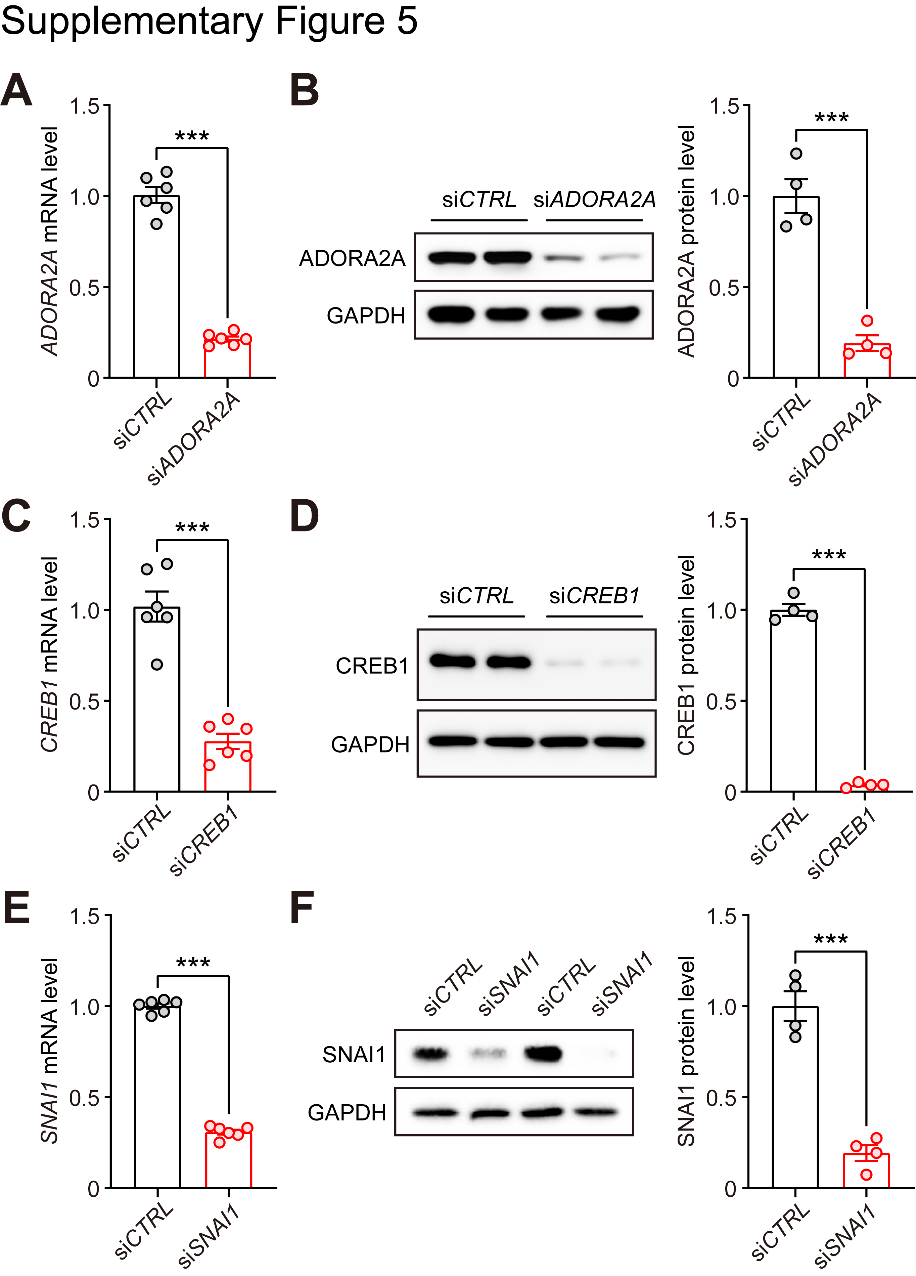
**

**Figure S5. Validation of siRNA knockdown efficiencies for ADORA2A, CREB1, and SNAI1 in HAECs. A,** Real‐time PCR analysis of mRNA expression for *ADORA2A* in HAECs exposed to si*ADORA2A* for 24 h (n = 6). **B,** Western blot analysis and quantification data of indicated protein level for ADORA2A in HAECs treated with si*ADORA2A* for 48 h (n = 4). **C,** Real‐time PCR analysis of mRNA expression for *CREB1* in HAECs exposed to si*CREB1* for 24 h (n = 6). **D,** Western blot analysis and quantification data of indicated protein level for CREB1 in HAECs treated with si*CREB1* for 48 h (n = 4). **E,** Real‐time PCR analysis of mRNA expression for *SNAI1* in HAECs exposed to si*SNAI1* for 24 h (n = 6). **F,** Western blot analysis and quantification data of indicated protein level for SNAI1 in HAECs treated with si*SNAI1* for 48 h (n = 4). Data are represented as means ± SEM. Statistical significance was determined by unpaired Student's *t*-test. ****p* < 0.001 for indicated comparisons.


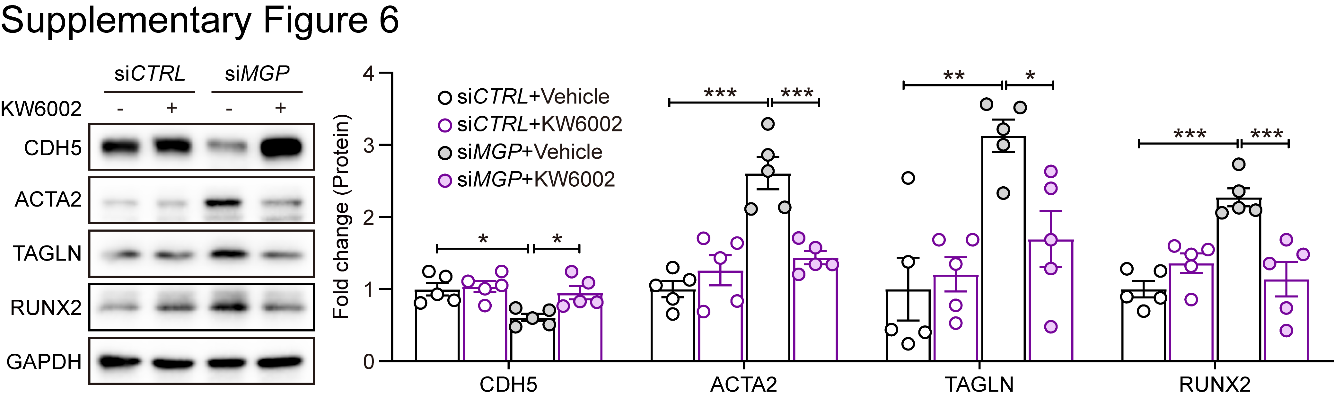
**Figure S6. Pharmacological inhibition of ADORA2A inhibits EndMT and osteogenesis of HAECs under pro-calcified conditions.** Western blot analysis and quantification data of indicated protein levels for endothelial marker (CDH5), mesenchymal markers (ACTA2 and TAGLN), and osteogenic marker (RUNX2) in *MGP* knockdown HAECs treated with vehicle or KW6002 treatment for 48 h (n = 5). Data are represented as means ± SEM. Statistical significance was determined by one-way ANOVA followed by Bonferroni’s *post hoc* test. **p* < 0.05, ***p* < 0.01, and ****p* < 0.001 for indicated comparisons.

**Supplemental Tables**

**Supplementary Table 1. Primers used for quantitative RT-PCR.**

| Gene | Forward (5’-3’) | Reverse (5’-3’) |
| --- | --- | --- |
| Murine *Adora2a* | TCCACTCCGGTACAATGGCTT | TGACTGCAGTTGTTCCAGCCC |
| Human *ADORA1* | TGCGAGTTCGAGAAGGTCATC | AGCTGCTTGCGGATTAGGTA |
| Human *ADORA2A* | CGAGGGCTAAGGGCATCATTG | CTCCTTTGGCTGACCGCAGTT |
| Human *ADORA2B* | CTCTTCCTCGCCTGCTTCGTG | TTATACCTGAGCGGGACACAG |
| Human *ADORA3* | TACATCATTCGGAACAAACTC | GTCTTGAACTCCCGTCCATAA |
| Human *CDH5* | GTTCACGCATCGGTTGTTCAA | CGCTTCCACCACGATCTCATA |
| Human *ACTA2* | TCCGCTTCAATTCCTGTCCG | CTTGATGCGAAGTGCTGACC |
| Human *TAGLN* | AGTGCAGTCCAAAATCGAGAAG | CTTGCTCAGAATCACGCCAT |
| Human *S100A4* | GTCCTGCATCGCCATGATGT | AACTTGCTCAGCATCAAGCAC |
| Human *RUNX2* | TAGGCGCATTTCAGGTGCTT | GACATGCCTGAGGTGACTGG |
| Human *MGP* | TCCGAGAACGCTCTAAGCCT | GCAAAGTCTGTAGTCATCACAGG |
| Human *FN1* | ACAACACCGAGGTGACTGAGAC | GGACACAACGATGCTTCCTGAG |
| Human *SNAI1* | TGCCCTCAAGATGCACATCCGA | GGGACAGGAGAAGGGCTTCTC |
| Human *CREB1* | CCACTGTAACGGTGCCAACT | GCTGCATTGGTCATGGTTAATGT |
| 18S rRNA | CTTAGAGGGACAAGTGGCG | ACGCTGAGCCAGTCAGTGTA |
